# Plasma Biomarker Profiling in Heart Failure Patients with Preserved Ejection Fraction before and after Spironolactone Treatment: Results from the Aldo-DHF Trial

**DOI:** 10.3390/cells10102796

**Published:** 2021-10-19

**Authors:** Moritz Schnelle, Andreas Leha, Abass Eidizadeh, Katharina Fuhlrott, Tobias D. Trippel, Djawid Hashemi, Karl Toischer, Rolf Wachter, Christoph Herrmann-Lingen, Gerd Hasenfuß, Burkert Pieske, Lutz Binder, Frank Edelmann

**Affiliations:** 1 Institute for Clinical Chemistry, University Medical Center Goettingen, 37075 Goettingen, Germany; moritz.schnelle@med.uni-goettingen.de (M.S.); abass.eidizadeh@med.uni-goettingen.de (A.E.); lutz.binder@med.uni-goettingen.de (L.B.); 2 DZHK (German Centre for Cardiovascular Research), Partner Site Goettingen, 37075 Goettingen, Germany; andreas.leha@med.uni-goettingen.de (A.L.); ktoischer@med.uni-goettingen.de (K.T.); rolf.wachter@medizin.uni-leipzig.de (R.W.); cherrma@gwdg.de (C.H.-L.); hasenfus@med.uni-goettingen.de (G.H.); 3 Department of Medical Statistics, University Medical Center Goettingen, 37075 Goettingen, Germany; 4 Clinic of Cardiology and Pneumology, University Medical Center Goettingen, 37075 Goettingen, Germany; katharinafuhlrott@yahoo.de; 5 Department of Internal Medicine and Cardiology, Charité-Universitätsmedizin Berlin, Campus Virchow Klinikum, 13353 Berlin, Germany; tobias_daniel.trippel@charite.de (T.D.T.); djawid.hashemi@charite.de (D.H.); burkert.pieske@charite.de (B.P.); 6 DZHK (German Centre for Cardiovascular Research), Partner Site Berlin, 10785 Berlin, Germany; 7 Clinic and Policlinic for Cardiology, University Hospital Leipzig, 04103 Leipzig, Germany; 8 Department of Psychosomatic Medicine and Psychotherapy, University Medical Center Goettingen, 37075 Goettingen, Germany; 9 Berlin Institute of Health, 13353 Berlin, Germany; 10 Department of Cardiology, Germany Heart Center Berlin, 13353 Berlin, Germany

**Keywords:** plasma biomarkers, heart failure with preserved ejection fraction, spironolactone

## Abstract

The pathophysiology of heart failure with preserved ejection fraction (HFpEF) is poorly understood and therapeutic strategies are lacking. This study aimed to identify plasma proteins with pathophysiological relevance in HFpEF and with respect to spironolactone-induced effects. We assessed 92 biomarkers in plasma samples from 386 HFpEF patients—belonging to the Aldo-DHF trial—before (baseline, BL) and after one-year treatment (follow up, FU) with spironolactone (verum) or a placebo. At BL, various biomarkers showed significant associations with the two Aldo-DHF primary end point parameters: 33 with E/e’ and 20 with peak VO_2_. Ten proteins including adrenomedullin, FGF23 and inflammatory peptides (e.g., TNFRSF11A, TRAILR2) were significantly associated with both parameters, suggesting a role in the clinical HFpEF presentation. For 13 proteins, expression changes from BL to FU were significantly different between verum and placebo. Among them were renin, growth hormone, adrenomedullin and inflammatory proteins (e.g., TNFRSF11A, IL18 and IL4RA), indicating distinct spironolactone-mediated effects. BL levels of five proteins, e.g., inflammatory markers such as CCL17, IL4RA and IL1ra, showed significantly different effects on the instantaneous risk for hospitalization between verum and placebo. This study identified plasma proteins with different implications in HFpEF and following spironolactone treatment. Future studies need to define their precise mechanistic involvement.

## 1. Introduction

Heart failure with preserved ejection fraction (HFpEF) imposes significant morbidity and mortality with a prevalence of up to 50% of all heart failure (HF) cases [1]. It is often associated with ageing, hypertension and/or obesity [2], yet the underlying mechanisms of this condition are still poorly understood. This may be why there has been only slight progress on novel, effective, therapeutic strategies to treat HFpEF [3]. Surprisingly, the use of β-adrenergic receptor blockers, prescribed to most HF patients, was recently shown to be associated with an increased risk of HF hospitalizations in HFpEF [4], highlighting the complex challenge to develop novel therapeutic strategies to treat this condition.

In the multicentre, prospective, randomized, double-blind, placebo-controlled Aldo-DHF study, spironolactone was tested as a potentially effective treatment for HFpEF [5]. This trial in 422 ambulatory HFpEF patients found, after one year of treatment with spironolactone, a reduction in left ventricular (LV) filling pressures (measured via echocardiography using E/e’ as a surrogate), LV mass and circulating amino-terminal pro-brain natriuretic peptide (NT-proBNP) concentrations. No significant changes in maximal exercise capacity or quality of life were observed. It is likely that spironolactone improves LV diastolic function via anti-fibrotic effects, as previously shown in experimental models using rats [6,7]. This is further supported by findings from the HOMAGE trial, which showed that spironolactone treatment reduced markers of collagen synthesis in patients at risk of developing HF [8], as well as findings from a recent biomarker study suggesting anti-fibrotic effects through spironolactone treatment in HFpEF patients with diabetes [9]. Our group recently demonstrated in the Aldo-DHF cohort that HFpEF patients with high levels of the carboxy-terminal telopeptide of collagen I to matrix metalloproteinase-1 ratio (CITP/MMP-1), a negative index of myocardial collagen cross-linking, particularly benefitted from spironolactone therapy [10]. It is therefore likely that spironolactone exerts its anti-fibrotic functions especially in HFpEF patients with lower degrees of collagen cross-linking in the heart.

The analysis of large panels of plasma or serum biomarkers in distinct patient cohorts is a common approach in cardiovascular science [11,12,13]. It can be used to identify underlying mechanisms of the disease of interest, or to analyse downstream effects of an administered drug. In this study, we measured 92 biomarkers (OLINK panel CVDII) in 386 plasma samples belonging to the Aldo-DHF trial at baseline and after twelve months of placebo or spironolactone treatment, respectively. The goals were to identify proteins/processes that are related to relevant clinical features (e.g., diastolic function, exercise capacity, cardiac remodelling and quality of life) and to discern possible spironolactone-induced effects in this well-characterized patient cohort.

## 2. Materials and Methods

The Aldo-DHF trial was a prospective, randomized, placebo-controlled, double-blind multicentre study, which assessed the efficacy of a long-term aldosterone receptor blockade through spironolactone treatment in HFpEF. The trial design and primary results of the Aldo-DHF study have been previously published [5]. Briefly, eligible patients were enrolled and randomized (1:1 ratio) to spironolactone 25 mg once daily or a matching placebo and followed up for twelve months. Patients with stable HFpEF defined as per the New York Heart Association (NYHA) class II or III HF symptoms, LV ejection fraction (LVEF) ≥50% at rest, echocardiographic evidence of grade  ≥ I diastolic dysfunction or atrial fibrillation and peak oxygen consumption ≤25 mL/kg/min were eligible for participation. Primary end points were changes in E/e’ (the ratio of peak early transmitral ventricular filling velocity to early diastolic tissue Doppler velocity as an echocardiographic estimate of left ventricular filling pressure) and peak exercise capacity (peak VO_2_ in cardiopulmonary exercise testing) at twelve months. Secondary end points included echocardiographic indices of cardiac remodelling and function (e.g., left ventricular mass index, LVMI, and left atrial volume index, LAVI), plasma levels of the N-terminal pro–brain-type natriuretic peptide (NT-proBNP, a marker for neuroendocrine activation) and quality of life assessment (e.g., through the SF-36 physical functioning scale score). Major exclusion criteria were prior-documented LVEF ≤40%, significant coronary artery disease, myocardial infarction or coronary artery bypass graft surgery within three months, definitive or probable pulmonary disease (vital capacity <80% or forced expiratory volume in 1 s (FEV_1_) <80% or reference values on spirometry), body mass index ≥36 kg/m^2^ or serum creatinine >1.8 mg/dL.

The Aldo-DHF study was conducted in accordance with national laws, guidelines for good clinical practice, as well as the Declaration of Helsinki. The study protocol was reviewed and approved by the institutional review board of each participating centre. All patients provided written informed consent prior to enrolment.

### 2.1. Plasma Biomarkers

In this study, plasma of 386 patients belonging to the Aldo-DHF cohort was assessed at baseline (BL) and twelve months following either placebo (placebo group; n = 187) or spironolactone (verum group; n = 199) treatment (follow up, FU). Patient characteristics at BL are reported in Supplementary Table S1. A total of 92 biomarkers were measured in heparin plasma using the Olink technology (Olink, Uppsala, Sweden). These markers were part of the pre-selected cardiovascular panel II (CVDII), which includes proteins with already-established associations with cardiovascular diseases as well as more exploratory ones. A full list of these biomarkers (including all abbreviations) can be found in Supplementary Table S2. The proteins were quantified using a high-throughput Olink Proseek Multiplex 96 × 96 kit, which measures all 92 proteins simultaneously in 1 μL of plasma. The kit uses a proximity extension assay (PEA) technology in which 92 oligonucleotide-labelled antibody probe pairs are allowed to bind to their respective targets in the sample. After several further steps, including a real-time polymerase chain reaction (a detailed description of the technique is provided on the company’s website https://www.olink.com/ accessed on 14 April 2021), log2-normalized protein expression (NPX) values were provided. These values correspond to the respective protein concentrations, but they do not represent absolute quantifications. Only samples passing the Olink quality control and measurements exceeding the respective limits of detection were included in our analysis.

The full list of protein expression levels in the different patient groups can be found in Supplementary Table S2.

### 2.2. Statistical Analysis

The BL expression of each biomarker was tested for its association with clinical parameters (e.g., E/e’, peak VO_2_) using linear models controlling for age and sex.

Each biomarker was tested for significant changes in time (i.e., BL to FU) separately within both study arms (i.e., verum vs. placebo) using linear mixed-effect regressions with the time point as covariate and controlling for age and sex. Additionally, each protein was tested for different time courses between the treatment arms using linear mixed-effect regression with time point in interaction with treatment arms as covariates and controlling for age and sex. Marginal means with 95% confidence intervals (CIs) were estimated and visualized as mean error plots overlaying box plots of the observed data.

Each biomarker was assessed for its potential influence on the time-to-first-hospitalization using Cox proportional-hazards regression models controlling for the study arm, age and sex. An interaction between the study arm and the respective protein was included if supported by likelihood ratio tests. For visualization purposes of this analysis, patients were split into two groups along the cut-off of the BL expression of the respective protein that maximizes the standardized log-rank statistic, and hospitalization-free survival probabilities were estimated using the Kaplan–Meier method. The resulting survival curves were plotted separately for both groups.

For each protein, the association of the respective BL expression with the change in time of the clinical parameters E/e’ and peak VO_2_ was assessed using linear models with the protein expression in interaction with the randomization arm as covariates and controlling for age and sex, and the BL value of the clinical parameter. For visualization purposes of this particular analysis, patients were split into three groups along the tertiles of the respective BL protein expression level. The clinical parameter was visualized separately by time point and study arm using line plots with overlaid boxplots.

For all regression analyses, the resulting regression coefficients (b-coefficients) were reported with 95% CIs and corresponding *p*-values against the null hypothesis of no association. *p*-values were adjusted for multiple testing across all proteins using the procedure of Benjamini–Hochberg. If not directly stated, data interpretation was based on analyses before multiple testing adjustments due to the explorative design of this study.

If not stated differently, the significance level was set to alpha = 5% for statistical tests. All analyses were performed with the statistical software R (version 3.6.1; R Core Team 2019 https://www.R-project.org/, accessed on 22 April 2021 ) using the R-package lme4 (version 1.1.21) [14] for the mixed-effect logistic regression. The degrees of freedom were estimated using Satterthwaite´s method as implemented in the ImerTest packages (version 3.1.0) [15]. Estimated marginal means were computed using the package emmeans (version 1.4.7).

## 3. Results

### 3.1. Baseline

In a first approach, we wanted to determine whether levels of plasma biomarkers correlated with BL E/e’ and peak VO_2_, respectively. Here, ten proteins were found to be significantly associated with both parameters: TNF-related apoptosis-inducing ligand receptor 2 (TRAILR2), fibroblast growth factor 23 (FGF23), tumour necrosis factor receptor superfamily member 11A (TNFRSF11A), adrenomedullin (ADM), hydroxyacid oxidase 1 (HAOX1), thrombospondin-2 (THBS2), mitochondrial carbonic anhydrase 5A (CA5A), angiotensin-converting enzyme 2 (ACE2), renin (REN) and spondin-2 (SPON2) (Table 1). TRAILR2, FGF23, TNFRSF11A and ADM remained significantly linked to E/e’ and peak VO_2_ even after adjusting for multiple testing (Table 1). The associations with E/e’ were positive and negative with respect to peak VO_2_, indicative of reduced diastolic function and exercise capacity when protein levels were relatively high. Expression levels of 23 additional proteins showed an association with E/e’, and 10 with peak VO_2_ only (Table 1). Levels of the brain natriuretic peptide (BNP) showed the most significant association (i.e., the lowest *p*-value) with E/e’, and levels of leptin (LEP) with peak VO_2_.

We also investigated the association between plasma biomarkers and secondary end points of the Aldo-DHF trial, i.e., the left ventricular mass index (LVMI, echocardiographic index of cardiac remodelling), plasma NT-proBNP concentrations (N-terminal pro-brain-type natriuretic peptide, a biomarker of cardiac stress released upon cardiac stretch) and quality of life using the SF-36 physical functioning scale score. Three proteins were found to be significantly associated with the LVMI (Supplementary Table S3a), 22 with NT-proBNP concentrations (Supplementary Table S3b) and 51 with the SF-36 score (Supplementary Table S3c). Interestingly, the previously mentioned ADM and FGF23 were also among the proteins with a positive association with plasma NT-proBNP concentrations and were negatively associated with the SF-36 score. Levels of the brain natriuretic peptide (BNP) were positively associated with the LVMI (Supplementary Table S3a), and as expected, with concentrations of its related peptide NT-proBNP (Supplementary Table S3b).

### 3.2. Effect of Spironolactone Treatment

**Figure 1:**
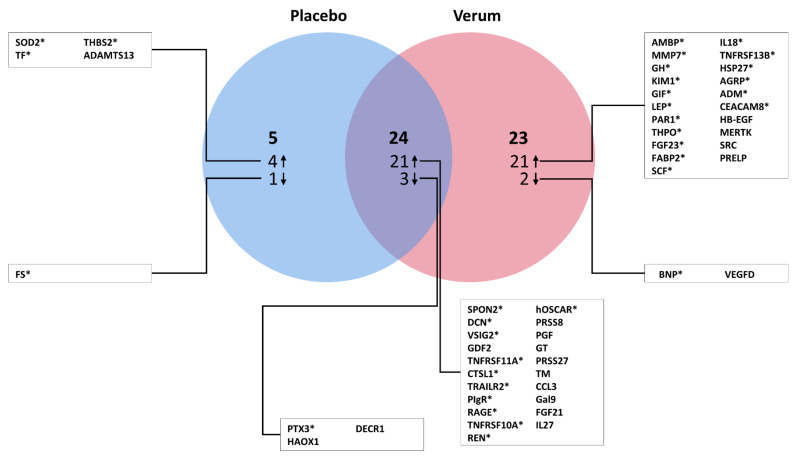
Expression changes of plasma biomarkers following spironolactone or placebo treatment in HFpEF. The Venn diagram shows plasma biomarkers with significant expression changes from baseline (BL) to twelve-month follow-up (FU) in the placebo group, the verum group and in both groups (corrected for age and sex). * indicates that statistical significance remains after adjustment for multiple testing (in the combined group, this implied that significance remained in both the placebo and verum groups).

**Figure 2:**
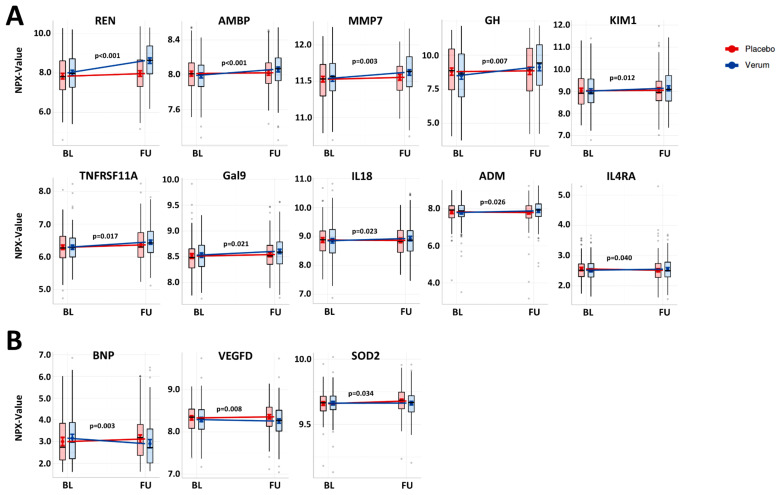
Effect of spironolactone treatment on differential regulation of plasma biomarkers in HFpEF. (**A**,**B**): Plasma biomarkers with significantly increased (**A**) or decreased (**B**) expression levels from baseline (BL) to twelve-month follow-up (FU) following spironolactone treatment (verum) vs. placebo control are shown (corrected for age and sex). The modelled effects are illustrated as expected marginal means with 95% CIs. The semi-transparent boxplots show the median (centre line) and the lower and the upper quartile (box) of the originally measured NPX values; the whiskers extend to the minimum/maximum, but are capped at a distance of 1.5 times the interquartile range above the upper/lower quartile. *p*-values of the interactions from linear mixed effect models are reported in the respective graphs.

Separate analysis of plasma biomarker expression level changes from BL to FU revealed marked differences between the placebo and verum groups. In the placebo group, only four proteins were upregulated and one downregulated, but in the verum groups, twenty-one were up- and two downregulated (Figure 1). Significant expression changes of 24 proteins (21 upregulated, 3 downregulated) were found to be similar in both treatment arms (Figure 1). The comparison of biomarker expression changes from BL to FU between both groups resulted in the identification of 13 proteins with significantly different regulation (Figure 2). Of these, ten proteins increased with spironolactone treatment vs. the placebo control: REN (b-coefficient: 0.49, 95%-CI: 0.35 to 0.63; *p* < 0.001), alpha-1-microglobulin/bikunin precursor (AMBP) (0.06, 0.03 to 0.09; *p* < 0.001), matrix metalloproteinase 7 (MMP7) (0.06, 0.02 to 0.10; *p* = 0.003), growth hormone (GH) (0.56, 0.15 to 0.98; *p* = 0.008), kidney injury molecule-1 (KIM1) (0.11, 0.03 to 0.20; *p* = 0.012), TNFRSF11A (0.08, 0.01 to 0.15; *p* = 0.017), galectin-9 (Gal9) (0.05, 0.01 to 0.08; *p* = 0.021), interleukin-18 (IL18) (0.09, 0.01 to 0.17; *p* = 0.023), ADM (0.12, 0.02 to 0.23; *p* = 0.026) and interleukin-4 receptor subunit alpha (IL4RA) (0.08, 0.00 to 0.15; *p* = 0.040). As compared to placebo controls, three proteins decreased following treatment with spironolactone vs. placebo: BNP (−0.37, −0.61 to −0.13; *p* = 0.003), vascular endothelial growth factor D (VEGFD) (−0.06, −0.11 to −0.02; *p* = 0.008) and the mitochondrial superoxide dismutase 2 (SOD2) (−0.02, −0.04 to −0.00; *p* = 0.034).

### 3.3. Prediction of Beneficial Effects through Spironolactone Treatment

**Figure 3:**
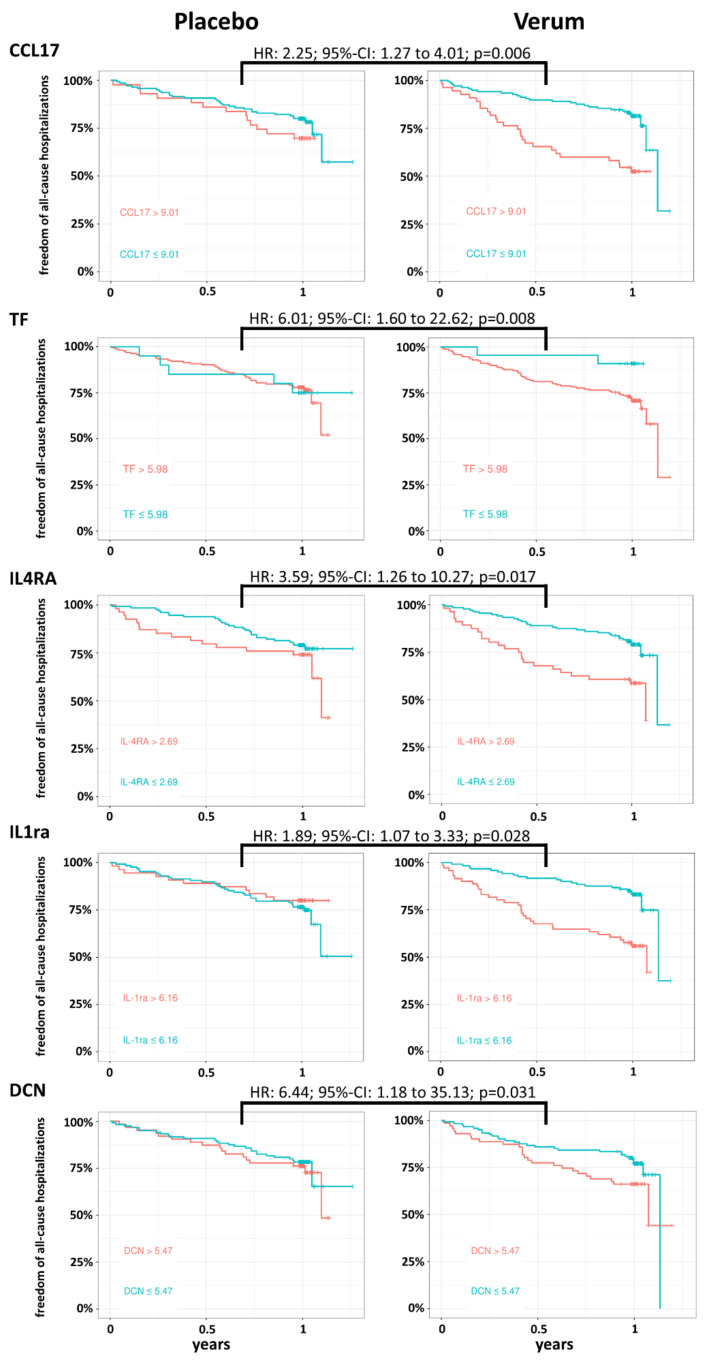
Plasma biomarkers with predictive value regarding spironolactone-mediated effects on all-cause hospitalizations in HFpEF patients. Based on data from the Cox proportional-hazards model (corrected for age and sex), Kaplan–Meier curves per study arm (spironolactone/verum vs. placebo) for the proteins with significant associations with the time-to-first-hospitalization are shown. For visualization purposes, the protein values are binarized at the cut-off of the respective BL expression level that maximizes the standardized log-rank statistic. The text annotation gives the hazards ratio (HR) with 95% CI and *p*-value of the interaction effect of BL expression and study arm.

We assessed whether BL expression levels of distinct plasma biomarkers could predict the effect of spironolactone treatment on (i) the freedom of all-cause hospitalizations and (ii) changes in E/e’ and peak VO_2_. Using a Cox proportional-hazards model for the time-to-first-hospitalization, we identified five proteins with significant interaction with the treatment arm (Figure 3). These proteins were the C-C motif chemokine ligand 17 (CCL17) (HR: 2.25, 95%-CI: 1.27 to 4.01; *p* = 0.006), tissue factor (TF) (6.01, 1.60 to 22.62; *p* = 0.008), IL4RA (3.59, 1.26 to 10.27; *p* = 0.017), interleukin-1 receptor antagonist (IL1ra) (1.89, 1.07 to 3.33; *p* = 0.028) and decorin (DCN) (6.44, 1.18 to 35.13; *p* = 0.031). For all of these proteins, lower BL values were generally associated with a reduced hazard for all-cause hospitalization in the verum group, but not in the placebo control cohort (Figure 3). The effect of the treatment with spironolactone on the relative change of the E/e’ ratio was significantly different from placebo controls according to the BL expression levels of three proteins: The C-C motif chemokine ligand 3 (CCL3) (b-coefficient: −0.09, 95%-CI: −0.17 to −0.02; *p* = 0.016), VEGFD (0.15, 0.02 to 0.28; *p* = 0.025) and pro-interleukin-16 (IL16) (−0.10, −0.19 to −0.01; *p* = 0.027) (Supplementary Figure S1). With respect to spironolactone-mediated changes of peak VO_2_, BL plasma levels of interleukin-18 (IL18) (−0.12, −0.20 to −0.04; *p* = 0.004), alpha-L-iduronidase (IDUA) (−0.11, −0.19 to −0.03; *p* = 0.007) and prostasin (PRSS8) (−0.13, −0.26 to 0.00; *p* = 0.043) were found to have predictive value (Supplementary Figure S2).

## 4. Discussion

To the best of our knowledge, this is the first report linking various circulating biomarkers to clinical parameters of diastolic function (i.e., echocardiographic E/e’), exercise capacity (i.e., peak VO_2_), cardiac remodelling (i.e., echocardiographic LVMI), neuroendocrine activation (i.e., plasma NT-proBNP concentrations) and quality of life (i.e., SF-36 physical functioning scale score) in a large cohort of HFpEF patients. We were able to demonstrate that BL plasma levels of several proteins were associated with these features, suggesting a potential role in HFpEF pathophysiology. Amongst these proteins, ADM and FGF23 could be identified as promising targets. Both showed positive associations with E/e’ and plasma NT-proBNP concentrations and were negatively associated with peak VO_2_ and the SF-36 score. High plasma levels of these proteins may therefore reflect enhanced diastolic dysfunction and neuroendocrine activation as well as reduced exercise capacity and quality of life. This is consistent with several previous reports showing that plasma ADM concentrations increase in HF and reflect the level of congestion [16,17]. Recently, ADM levels were demonstrated to be elevated in HFpEF patients, highlighting the potential relevance of this vasodilatory peptide hormone in this pathological condition [18]. Using experimental models, FGF23 was shown to augment the development of LV hypertrophy and exacerbate diastolic dysfunction through the induction of cardiac fibrosis [19,20]. Based on these findings and our observations, FGF23 may be a key driver of clinical deterioration in HFpEF. This hypothesis is also supported by a recent study showing that higher plasma levels of FGF23 were linked to incident HFpEF rather than HFrEF [21]. Future studies need to define the precise roles of ADM and FGF23 in the pathophysiology of HFpEF.

Levels of REN were associated with E/e’ and peak VO_2_, emphasizing the importance of the renin–angiotensin–aldosterone system in HFpEF. Blockade of this system, e.g., through spironolactone treatment as conducted in the Aldo-DHF trial [5], therefore appears to be a promising therapeutic strategy in HFpEF. We also found plasma levels of BNP to be significantly associated with E/e’. A similar observation was recently shown for NT-proBNP in the Aldo-DHF cohort [22]. The assessment of plasma BNP concentrations plays a central role in the HFpEF diagnostic algorithm [23]. Accordingly, it was previously demonstrated that plasma BNP concentrations correlate with the degree of end-diastolic wall stress in patients with diastolic HF [24]. We now provide further evidence of the direct, positive link between plasma BNP levels and E/e’ in HFpEF. The associations of E/e’ and peak VO_2_ with TRAILR2 and TNFRSF11A indicate that inflammatory processes appear to be relevant in this context. The value of TRAILR2 as a cardiovascular biomarker was recently demonstrated as plasma levels could be linked to incident HF in elderly individuals [25]. In addition to TRAILR2 and TNFSRF11A, we found a high number of other proteins related to inflammatory processes with significant associations with E/e’ or peak VO_2_ (e.g., IL6, IL 18, IL27, MARCO, XCL1), but also with plasma NT-proBNP concentrations and/or the SF-36 physical functioning scale score (e.g., IL4RA, IL17D, MARCO, PTX3, TRAILR2). Inflammation is a pathophysiological hallmark of HFpEF [26]. Accordingly, Tromp et al. recently demonstrated in a plasma biomarker approach (similar to ours) that inflammatory processes appear to be more dominant features of HFpEF as compared to HFrEF [11]. Our findings add to this observation, as various inflammatory proteins in HFpEF patients could be directly linked to relevant clinical features. Additionally, different markers related to metabolism and extracellular matrix (ECM) organization revealed significant associations with clinical readouts (e.g., LEP, DECR1, LPL, DCN, MMP7, MMP12). Remarkably, levels of LEP showed the most significant association with peak VO_2_. This is in line with the general belief that HFpEF is a heterogenous disease encompassing several different underlying mechanisms, including metabolic and ECM organization-related processes [26,27].

Spironolactone is known to reduce morbidity and mortality in patients with severe HFrEF [28]. It exerts its beneficial effects through mineralocorticoid receptor (MR) blockade and may thereby attenuate maladaptive downstream effects through aldosterone-mediated MR activation, e.g., increases in reactive oxygen species, inflammation, fibrosis, mitochondrial dysfunction and adrenergic receptor modulation [29,30]. In the Aldo-DHF trial, distinct beneficial effects through spironolactone treatment in HFpEF patients were demonstrated including an improvement of diastolic function and neuroendocrine activation, as well as a reduction of adverse cardiac remodelling [5]. The TOPCAT trial, however, reported no beneficial effects through spironolactone treatment in HFpEF patients with respect to cardiovascular mortality and HF hospitalization [31]. However, regional variations in the TOPCAT trial appear to be critical, as specifically HFpEF patients from the Americas (United States, Canada, Brazil, Argentina) showed a possible clinical benefit through MR blockade [32]. Thus, the value of spironolactone or other MR antagonists in HFpEF is still under debate, and this may even differ between distinct HFpEF phenotypes [33]. Yet there is increasing evidence from several recent reports that MR blockade exerts favourable effects in HFpEF and should therefore be used in the treatment of this pathological condition [34,35,36,37,38,39,40]. To gain further insights, we evaluated spironolactone-mediated effects in the Aldo-DHF HFpEF cohort. The upregulation of REN reflected sufficient blockade of the MR, and the reduction of BNP is in line with the decreased levels of its related peptide NT-proBNP as shown in the original Aldo-DHF study [5]. Plasma levels of the two hormones ADM (see discussion above) and GH were increased following treatment with spironolactone. Both hormones are believed to play protective roles in HF through different mechanisms, including anti-fibrotic effects [41,42,43,44]. It may therefore be speculated that spironolactone mediates distinct beneficial effects in HFpEF by enhancing ADM and GH expression. The increase in MMP7 plasma levels observed in our study is consistent with recent findings demonstrating a similar regulation through spironolactone in a cohort of patients at risk of developing HF (HOMAGE trial) [12]. Interestingly, plasma levels of MMP7 were also reported to be increased in patients with diastolic HF [45]. Since MMP7 was shown (i) to have a detrimental role in cardiac remodelling post myocardial infarction (MI) and (ii) to promote the development of renal fibrosis in experimental mouse models [46,47], it remains to be seen whether the spironolactone-induced increase in MMP7 expression may also cause damage in the HFpEF setting. However, it is likely that the function of MMP7 may differ depending on the underlying pathophysiology. Gal9 levels were also found to be increased in the verum group. Gal9 may be involved in immunological regulations and have anti-atherosclerotic properties [48]. Galectin-3, a family member of Gal9, is a marker of cardiac fibrosis and was recently shown to be associated with worse outcomes in the Aldo-DHF cohort [22]. Other biomarkers related to inflammation were also increased in the verum group (i.e., TNFRSF11A, IL18 and IL4RA), suggesting that spironolactone induces distinct inflammatory changes. A similar finding was recently reported in the HOMAGE cohort, in which increased plasma levels of IL12B, IL6RA and TNFRSF9 following spironolactone treatment as compared to controls were observed [12]. Previous studies using different cardiovascular disease models have demonstrated that the MR on macrophages/monocytes is a key regulator of cardiac remodelling [49,50,51]. We hypothesize that the spironolactone-induced effects observed here on inflammatory markers may also be due to myeloid cell MR modulation. To further investigate this, detailed assessment of an HFpEF mouse model with myeloid cell MR deletion would be an interesting approach. Levels of VEGFD are reduced in both the Aldo-DHF and the HOMAGE cohorts, indicating a modulatory role of spironolactone in angiogenesis. As these changes could be observed in two different patient cohorts, they may reflect general spironolactone-induced effects independent of the underlying pathophysiology. The reduced levels of SOD2 may be attributed to the decrease in oxidative stress through spironolactone [52], resulting in a lower demand for superoxide dismutation.

This study further demonstrated that BL expression levels of distinct proteins, including several markers related to inflammation, may predict the effects of spironolactone on freedom from all-cause hospitalizations as well as changes of E/e’ or peak VO_2_. In this regard, our data suggest that beneficial effects through spironolactone may depend on levels of distinct inflammatory cytokines (e.g., interleukins and related peptides such as IL4RA, IL1ra, IL16 and IL18) and chemokines (e.g., CCL17, CCL3), a group of molecules known to be involved in HF development [53]. Accordingly, IL16 was shown to promote cardiac fibrosis and myocardial stiffening in HFpEF, and IL18 was shown to induce cardiac dysfunction including diastolic impairment [54,55]. CCL17, a pro-fibrotic chemokine produced by activated M2 macrophages [56], was shown to be increased in blood samples of a small HFpEF cohort (n = 30) [57]. Of note, CCL17 and IL4RA are mechanistically linked since IL4RA signalling is involved in M2 macrophage polarization [58]. Taken together, these findings suggest that spironolactone-mediated effects in HFpEF may be dependent on distinct patients’ inflammatory signatures.

### Study Limitations

(i) As this is an explorative study, unless stated otherwise, data interpretation was generally based on statistical analyses before adjustment for multiple testing. This increases the chance of obtaining false-positive results. (ii) The analysis included measurements of 92 pre-selected biomarkers (CVDII panel by OLINK); a future approach including more proteins, e.g., different collagens or other fibrosis-associated proteins, would provide more detailed information. In addition, a completely unbiased approach without pre-selection would enable screening for functional enrichment. (iii) Analyses in plasma, as carried out in this study, may not necessarily specifically reflect processes in the heart but may be affected by other organs. (iv) Consequently, this study design allows one to generate hypotheses, but not causal relationships between protein expression and clinical features.

## 5. Conclusions

To our knowledge, this is the first study linking expression levels of a large panel of plasma biomarkers to distinct clinical features of diastolic function and exercise capacity in HFpEF patients. Out of 92 biomarkers, we could identify ten to be associated with both E/e’ and peak VO_2_. These could be related to inflammation, fibrosis and the endocrine system. Our plasma biomarker approach was additionally used to gain more insights into how spironolactone may exert downstream effects in HFpEF. Several proteins with different implications could be identified, e.g., ADM and various peptides of inflammation including different cytokines and chemokines. Our findings suggest that the modulation of inflammatory processes could be a promising therapeutic strategy for HFpEF, but future studies are needed to better understand their mechanistic role.

## Figures and Tables

**Table 1:** Significant associations between biomarker expression levels and E/e’ and/or peak VO_2_ in HFpEF patients at baseline.

Biomarker	E/e’		Peak VO_2_ [mL/min/kg]	
	b-Coefficient (95%-CI)	*p*-Value	b-Coefficient (95%-CI)	*p*-Value
Significant association with E/e’ and peak VO_2_				
**TRAILR2**	1.55 (0.57 to 2.52)	0.002 *	−1.35 (−2.18 to −0.52)	0.001 *
**FGF23**	0.97 (0.33 to 1.61)	0.003 *	−0.86 (−1.41 to −0.32)	0.002 *
**TNFRSF11A**	1.23 (0.37 to 2.09)	0.005 *	−1.29 (−2.02 to −0.57)	0.001 *
**ADM**	0.90 (0.24 to 1.57)	0.008 *	−1.52 (−2.08 to −0.96)	<0.001 *
**HAOX1**	0.56 (0.30 to 0.82)	<0.001 *	−0.29 (−0.51 to −0.07)	0.011
**THBS2**	3.31 (1.11 to 5.51)	0.003 *	−2.39 (−4.26 to −0.52)	0.012
**CA5A**	0.77 (0.35 to 1.19)	<0.001 *	−0.35 (−0.70 to −0.01)	0.044
**ACE2**	0.74 (0.14 to 1.33)	0.015	−0.71 (−1.21 to −0.21)	0.006
**REN**	0.48 (0.10 to 0.85)	0.013	−0.35 (−0.67 to −0.04)	0.029
**SPON2**	2.73 (0.32 to 5.14)	0.026	−2.35 (−4.39 to −0.32)	0.024
Significant association with E/e’ only				
**BNP**	1.12 (0.64 to 1.60)	<0.001 *	−0.11 (−0.47 to 0.25)	0.542
**DECR1**	0.64 (0.29 to 0.99)	<0.001 *	−0.00 (−0.30 to 0.30)	0.996
**CTSL1**	1.92 (0.85 to 2.98)	<0.001 *	0.19 (−0.72 to 1.11)	0.680
**KIM1**	0.83 (0.36 to 1.30)	0.001 *	−0.39 (−0.79 to 0.02)	0.061
**IL27**	2.15 (0.93 to 3.38)	0.001 *	0.20 (−0.86 to 1.25)	0.715
**MARCO**	3.13 (1.33 to 4.92)	0.001 *	−0.43 (−1.97 to 1.11)	0.586
**FS**	1.12 (0.47 to 1.76)	0.001 *	−0.14 (−0.69 to 0.42)	0.630
**XCL1**	1.11 (0.44 to 1.79)	0.001 *	−0.44 (−1.02 to −0.14)	0.137
**SORT1**	2.34 (0.91 to 3.78)	0.001 *	−0.28 (−1.51 to 0.95)	0.658
**FABP2**	0.81 (0.31 to 1.30)	0.001 *	0.33 (−0.09 to 0.75)	0.126
**IL18**	1.01 (0.32 to 1.69)	0.004 *	0.01 (−0.58 to 0.60)	0.975
**MERTK**	1.50 (0.48 to 2.53)	0.004 *	0.04 (−0.83 to 0.92)	0.924
**GT**	0.91 (0.28 to 1.54)	0.005 *	0.32 (−0.22 to 0.86)	0.245
**CCL3**	0.84 (0.23 to 1.44)	0.007 *	−0.45 (−0.97 to 0.06)	0.083
**HO1**	1.15 (0.30 to 2.00)	0.008 *	0.13 (−0.59 to 0.86)	0.721
**CD4**	1.74 (0.42 to 3.05)	0.010 *	−0.92 (−2.04 to 0.20)	0.106
**MMP7**	1.73 (0.32 to 3.14)	0.017	−1.08 (−2.28 to 0.12)	0.078
**DCN**	2.09 (0.35 to 3.83)	0.019	0.73 (−0.75 to 2.22)	0.331
**PRELP**	2.30 (0.32 to 4.29)	0.023	−1.11 (−2.80 to 0.58)	0.197
**SLAMF7**	0.66 (0.08 to 1.24)	0.027	0.09 (−0.40 to 0.59)	0.714
**AGRP**	1.23 (0.13 to 2.33)	0.029	0.15 (−0.79 to 1.08)	0.761
**TNFRSF13B**	1.12 (0.10 to 2.15)	0.032	−0.46 (−1.33 to 0.42)	0.305
**RAGE**	1.01 (0.04 to 1.97)	0.041	−0.24 (−1.06 to 0.58)	0.570
Significant association with peak VO_2_ only				
**LEP**	0.47 (−0.01 to 0.94)	0.054	−1.44 (−1.81 to −1.06)	<0.001 *
**IL1ra**	0.33 (−0.22 to 0.87)	0.240	−1.17 (−1.62 to −0.73)	<0.001 *
**Gal9**	1.13 (−0.15 to 2.40)	0.082	−2.25 (−3.31 to −1.19)	<0.001 *
**FGF21**	0.19 (−0.09 to 0.47)	0.181	−0.41 (−0.64 to −0.18)	0.001 *
**IL6**	0.43 (−0.04 to 0.89)	0.070	−0.54 (−0.93 to −0.15)	0.007
**PSGL1**	1.17 (−0.27 to 2.60)	0.111	−1.57 (−2.77 to −0.36)	0.011
**PDGF subunit B**	0.32 (−0.08 to 0.72)	0.113	0.40 (0.06 to 0.73)	0.021
**CXCL1**	0.16 (−0.37 to 0.69)	0.561	0.50 (0.05 to 0.95)	0.028
**VSIG2**	0.22 (−0.41 to 0.84)	0.490	−0.55 (−1.08 to −0.03)	0.039
**ANGPT1**	0.10 (−0.38 to 0.58)	0.682	0.43 (0.02 to 0.83)	0.041

Linear regression analyses between plasma biomarkers and E/e’ and peak VO_2_ including age and sex as co-variables were conducted. Proteins with significant associations are ordered by significance in the respective sections; * indicates that statistical significance remains after adjustment for multiple testing.

## Data Availability

Data are stored at the University Medical Center Goettingen and can be made available upon reasonable request by M.S.

## Supplementary Materials

The following are available online at https://www.mdpi.com/article/10.3390/cells10102796/s1, Supplementary Table S1: Baseline characteristics. Supplementary Table S2: Full list of plasma biomarker expression levels. Supplementary Table S3a: Significant associations between the left ventricular mass index (LVMI) and plasma biomarker levels in HFpEF patients at baseline. Supplementary Table S3b: Significant associations between plasma NT-proBNP concentrations and plasma biomarker levels in HFpEF patients at baseline. Supplementary Table S3c: Significant associations between SF-36 physical functioning scale score and plasma biomarker levels in HFpEF patients at baseline. Supplementary Figure S1: Plasma biomarkers with predictive value in regard to spironolactone-mediated effects on relative E/e’ changes in HFpEF patients. Supplementary Figure S2: Plasma biomarkers with predictive value in regard to spironolactone-mediated effects on relative peak VO_2_ changes in HFpEF patients.
