# Supplementary material for: Plasma Biomarker Profiling in Heart Failure Patients with Preserved Ejection Fraction before and after Spironolactone Treatment: Results from the Aldo-DHF Trial
Source: Cells. 2021 Oct 19;10(10):2796. doi: 10.3390/cells10102796 (PMC8535031; doi:10.3390/cells10102796)
Supplement: Supplementary file 1 [file cells-10-02796-s001.zip › cells-1402460-supplementary.pdf]

SUPPLEMENTARY MATERIALS

Plasma biomarker profiling in heart failure patients with preserved ejection fraction before and after spironolactone treatment: results from the Aldo-DHF trial

by Schnelle et al.

TABLE OF CONTENTS

Suppl. Table S1: Baseline characteristics.

Suppl. Table S2: Full list of plasma biomarker expression levels.

Suppl. Table S3a: Significant associations between the left ventricular mass index (LVMI) and plasma biomarker levels in HFpEF patients at baseline.

Suppl. Table S3b: Significant associations between plasma NT-proBNP concentrations and plasma biomarker levels in HFpEF patients at baseline.

Suppl. Table S3c: Significant associations between SF-36 physical functioning scale score and plasma biomarker levels in HFpEF patients at baseline.

Suppl. Figure S1: Plasma biomarkers with predictive value in regard to spironolactone-mediated effects on relative E/e' changes in HFpEF patients.

Suppl. Figure S2: Plasma biomarkers with predictive value in regard to spironolactone-mediated effects on relative peak VO_2 changes in HFpEF patients.

Suppl. Table S1: Baseline characteristics.

	Total (n = 386)	Placebo (n = 187)	Verum (n = 199)
**Demographics**			
Male gender, n (%)	183 (47.4%)	92 (49.2%)	91 (45.7%)
Age, years	67.3 ± 7.6	67.3 ± 7.6	67.4 ± 7.6
**Medical history, n (%)**			
Coronary artery disease	151 (39.1%)	68 (36.4%)	83 (41.7%)
Hypertension	353 (91.5%)	170 (90.9%)	183 (92.0%)
Hyperlipidaemia	229 (59.3%)	120 (64.2%)	109 (54.8%)
Diabetes mellitus	66 (17.1%)	32 (17.1%)	34 (17.1%)
**Physical examination (n)**			
SBP, mmHg	135.6 ± 17.8 (385)	135.9 ± 18.1 (187)	135.3 ± 17.6 (198)
DBP, mmHg	79.3 ± 10.9 (385)	79.5 ± 11.7 (187)	79.1 ± 10.1 (198)
HR, b.p.m.	66.6 ± 11.1 (385)	65.8 ± 10.9 (187)	67.4 ± 11.2 (198)
**Laboratory measures (n)**			
Haemoglobin, g/dL	13.8 ± 1.2 (384)	13.8 ± 1.2 (187)	13.8 ± 1.2 (197)
Creatinine, mg/dL	0.9 ± 0.2 (385)	0.9 ± 0.2 (187)	0.9 ± 0.2 (198)
NT-proBNP, ng/L; median, IQR	159.2, 84.3-305.7 (375)	147.8, 83.7-276.0 (183)	180.7, 85.0-313.3 (192)
**Echocardiography (n)**			
LVEF, %	67.5 ± 7.8 (386)	67.9 ± 7.5 (187)	67.2 ± 8.0 (199)
LVMI, g/m²	106.8 ± 28.1 (386)	107.8 ± 26.9 (187)	105.9 ± 29.3 (199)
E/e', ratio	12.8 ± 3.9 (385)	12.8 ± 4.2 (186)	12.8 ± 3.6 (199)
**Cardiopulmonary exercise testing (n)**			
Peak VO_2, mL/min/kg	16.3 ± 3.5 (386)	16.3 ± 3.4 (187)	16.3 ± 3.6 (199)

Values are expressed as mean ± standard deviation or median and interquartile range (IQR), and categorical variables as number (%).

DBP: diastolic blood pressure; E/e': ratio of peak early transmitral ventricular filling velocity to early diastolic tissue Doppler velocity; HR: heart rate; LVEF: left ventricular ejection fraction; LVMI: left ventricular mass index; SBP: systolic blood pressure.

Suppl. Table S2: Full list of plasma biomarker expression levels. Shown are NPX values at baseline (BL) and following twelve months (follow up, FU) of spironolactone (verum) or placebo treatment.

Biomarker	Total	BL	FU	Placebo BL	Placebo FU	Verum BL	Verum FU
**n**	772	386	386	187	187	199	199
**BONE MORPHOGENETIC PROTEIN 6 (BMP6)**							
mean ± sd	3.66 ± 0.70	3.67 ± 0.70	3.66 ± 0.70	3.67 ± 0.70	3.70 ± 0.60	3.66 ± 0.80	3.62 ± 0.80
median (min; max)	3.76 (1.08; 7.04)	3.79 (1.08; 6.88)	3.74 (1.15; 7.04)	3.78 (1.08; 6.88)	3.76 (1.41; 7.04)	3.79 (1.13; 5.30)	3.73 (1.15; 5.72)
**ANGIOPOIETIN-1 (ANGPT1)**							
mean ± sd	7.19 ± 0.80	7.16 ± 0.80	7.22 ± 0.80	7.13 ± 0.80	7.20 ± 0.80	7.18 ± 0.80	7.25 ± 0.80
median (min; max)	7.07 (5.44; 10.25)	7.03 (5.44; 10.25)	7.09 (5.52; 9.97)	7.01 (5.63; 10.25)	7.02 (5.64; 9.97)	7.08 (5.44; 9.84)	7.21 (5.52; 9.43)
**ADRENOMEDULLIN (ADM)**							
mean ± sd	7.83 ± 0.60	7.81 ± 0.60	7.85 ± 0.60	7.83 ± 0.60	7.79 ± 0.70	7.79 ± 0.60	7.90 ± 0.60
median (min; max)	7.91 (3.16; 9.26)	7.92 (3.50; 9.01)	7.91 (3.16; 9.26)	7.94 (4.15; 9.01)	7.87 (3.16; 9.23)	7.89 (3.50; 8.99)	7.94 (4.89; 9.26)
**CD40 LIGAND (CD40L)**							
mean ± sd	4.29 ± 0.80	4.31 ± 0.80	4.28 ± 0.90	4.31 ± 0.80	4.27 ± 0.90	4.30 ± 0.80	4.30 ± 0.90
median (min; max)	4.16 (2.17; 8.15)	4.16 (2.67; 8.15)	4.15 (2.17; 7.61)	4.16 (2.67; 8.15)	4.13 (2.17; 7.61)	4.16 (2.84; 7.03)	4.19 (2.46; 6.85)
**SLAM FAMILY MEMBER 7 (SLAMF7)**							

mean ± sd	4.42 ± 0.70	4.41 ± 0.70	4.44 ± 0.70	4.40 ± 0.70	4.44 ± 0.70	4.42 ± 0.60	4.44 ± 0.70
median (min; max)	4.35 (2.77; 7.04)	4.35 (2.77; 6.90)	4.37 (2.86; 7.04)	4.32 (2.88; 6.90)	4.38 (3.01; 7.02)	4.38 (2.77; 6.61)	4.36 (2.86; 7.04)
**PLACENTA GROWTH FACTOR (PGF)**							
mean ± sd	9.12 ± 0.40	9.09 ± 0.40	9.16 ± 0.40	9.08 ± 0.40	9.14 ± 0.40	9.10 ± 0.40	9.18 ± 0.40
median (min; max)	9.11 (7.95; 10.42)	9.09 (8.11; 10.35)	9.13 (7.95; 10.42)	9.09 (8.18; 10.30)	9.12 (7.95; 10.32)	9.10 (8.11; 10.35)	9.18 (8.40; 10.42)
**A DISINTEGRIN AND METALLOPROTEINASE WITH THROMBOSPONDIN TYPE 1 REPEATS 13 (ADAMTS13)**							
mean ± sd	7.78 ± 0.10	7.78 ± 0.10	7.79 ± 0.10	7.78 ± 0.10	7.80 ± 0.10	7.78 ± 0.10	7.78 ± 0.20
median (min; max)	7.78 (7.15; 8.29)	7.78 (7.29; 8.16)	7.79 (7.15; 8.29)	7.78 (7.29; 8.13)	7.79 (7.40; 8.29)	7.77 (7.30; 8.16)	7.79 (7.15; 8.25)
**BROTHER OF CD0 (BOC)**							
mean ± sd	4.98 ± 0.00	4.98 ± 0.00	4.99 ± 0.00	4.99 ± 0.00	5.00 ± 0.00	4.98 ± 0.00	4.97 ± 0.00
median (min; max)	4.99 (4.06; 5.79)	4.99 (4.06; 5.60)	4.99 (4.18; 5.79)	4.99 (4.29; 5.56)	4.99 (4.18; 5.77)	4.99 (4.06; 5.60)	4.99 (4.28; 5.79)
**INTERLEUKIN-4 RECEPTOR SUBUNIT ALPHA (IL4RA)**							
mean ± sd	2.54 ± 0.40	2.54 ± 0.40	2.54 ± 0.40	2.56 ± 0.40	2.52 ± 0.40	2.52 ± 0.40	2.55 ± 0.40
median (min; max)	2.51 (1.56; 5.30)	2.51 (1.65; 5.29)	2.51 (1.56; 5.30)	2.52 (1.75; 5.29)	2.51 (1.62; 5.30)	2.50 (1.65; 3.67)	2.51 (1.56; 3.70)
**PROTO-ONCOGENE TYROSINE-PROTEIN KINASE SRC (SRC)**							

mean ± sd	6.32 ± 1.00	6.26 ± 0.90	6.38 ± 1.00	6.25 ± 0.90	6.30 ± 1.00	6.27 ± 0.90	6.45 ± 1.00
median (min; max)	6.25 (2.92; 9.02)	6.23 (4.06; 9.02)	6.34 (2.92; 8.39)	6.23 (4.06; 8.39)	6.24 (2.92; 8.39)	6.21 (4.11; 9.02)	6.44 (3.34; 8.37)
**INTERLEUKIN-1 RECEPTOR ANTAGONIST (IL1ra)**							
mean ± sd	5.97 ± 1.00	5.97 ± 1.00	5.97 ± 1.00	5.92 ± 0.70	5.90 ± 0.60	6.01 ± 1.00	6.04 ± 1.00
median (min; max)	5.89 (4.47; 8.36)	5.88 (4.47; 8.19)	5.90 (4.57; 8.36)	5.81 (4.47; 8.13)	5.89 (4.57; 7.89)	5.94 (4.56; 8.19)	5.90 (4.60; 8.36)
**INTERLEUKIN-6 (IL6)**							
mean ± sd	4.49 ± 0.90	4.46 ± 0.80	4.51 ± 0.90	4.45 ± 0.80	4.46 ± 0.90	4.47 ± 0.90	4.56 ± 0.90
median (min; max)	4.35 (2.82; 9.71)	4.34 (2.82; 8.59)	4.36 (2.96; 9.71)	4.34 (2.82; 7.55)	4.32 (2.96; 9.71)	4.38 (2.85; 8.59)	4.40 (3.15; 8.68)
**TUMOR NECROSIS FACTOR RECEPTOR SUPERFAMILY MEMBER 10A (TNFRSF10A)**							
mean ± sd	3.90 ± 0.50	3.86 ± 0.50	3.94 ± 0.50	3.82 ± 0.40	3.87 ± 0.40	3.90 ± 0.50	4.00 ± 1.00
median (min; max)	3.87 (2.86; 8.77)	3.84 (2.99; 8.77)	3.93 (2.86; 8.61)	3.80 (3.00; 4.82)	3.88 (2.86; 5.37)	3.84 (2.99; 8.77)	3.97 (3.03; 8.61)
**SERIN/THREONINE PROTEIN KINASE 4 (STK4)**							
mean ± sd	3.22 ± 0.90	3.21 ± 0.90	3.23 ± 0.80	3.19 ± 0.80	3.23 ± 0.90	3.22 ± 0.90	3.23 ± 0.80
median (min; max)	3.02 (2.12; 7.53)	3.02 (2.12; 7.53)	3.02 (2.13; 6.35)	3.01 (2.12; 6.51)	3.03 (2.13; 6.35)	3.05 (2.12; 7.53)	3.02 (2.14; 5.65)
**ALPHA-L-IDURONIDASE (IDUA)**							

mean ± sd	6.28 ± 0.50	6.26 ± 0.60	6.29 ± 0.50	6.27 ± 0.60	6.28 ± 0.50	6.26 ± 0.60	6.30 ± 0.50
median (min; max)	6.31 (3.30; 7.57)	6.30 (3.30; 7.47)	6.32 (4.11; 7.57)	6.28 (3.92; 7.47)	6.31 (4.11; 7.57)	6.34 (3.30; 7.36)	6.32 (4.58; 7.52)
**TUMOR NECROSIS FACTOR RECEPTOR SUPERFAMILY MEMBER 11A (TNFRSF11A)**							
mean ± sd	6.35 ± 0.50	6.29 ± 0.50	6.41 ± 0.50	6.29 ± 0.50	6.36 ± 0.50	6.29 ± 0.50	6.45 ± 0.50
median (min; max)	6.31 (5.12; 8.24)	6.28 (5.14; 8.23)	6.39 (5.12; 8.24)	6.28 (5.14; 8.05)	6.31 (5.24; 8.24)	6.28 (5.31; 8.23)	6.43 (5.12; 7.82)
**PROTEINASE-ACTIVATED RECEPTOR 1 (PAR1)**							
mean ± sd	8.55 ± 0.40	8.51 ± 0.40	8.60 ± 0.40	8.50 ± 0.40	8.56 ± 0.40	8.52 ± 0.40	8.64 ± 0.40
median (min; max)	8.57 (6.08; 10.02)	8.53 (6.15; 9.84)	8.60 (6.08; 10.02)	8.53 (7.33; 9.34)	8.57 (6.83; 9.69)	8.53 (6.15; 9.84)	8.65 (6.08; 10.02)
**TNF-RELATED APOPTOSIS-INDUCING LIGAND RECEPTOR 2 (TRAILR2)**							
mean ± sd	6.55 ± 0.40	6.50 ± 0.40	6.59 ± 0.40	6.50 ± 0.40	6.56 ± 0.40	6.51 ± 0.40	6.63 ± 0.40
median (min; max)	6.51 (5.41; 8.27)	6.47 (5.46; 8.15)	6.56 (5.41; 8.27)	6.46 (5.62; 7.91)	6.50 (5.60; 8.11)	6.47 (5.46; 8.15)	6.59 (5.41; 8.27)
**SERIN PROTEASE 27 (PRSS27)**							
mean ± sd	9.64 ± 0.50	9.61 ± 0.50	9.67 ± 0.50	9.58 ± 0.40	9.63 ± 0.50	9.63 ± 0.50	9.70 ± 0.50
median (min; max)	9.60 (8.22; 11.87)	9.57 (8.51; 11.52)	9.64 (8.22; 11.87)	9.56 (8.53; 10.71)	9.63 (8.22; 10.78)	9.57 (8.51; 11.52)	9.64 (8.75; 11.87)
**ANGIOPOIETIN-1 RECEPTOR (TIE2)**							

mean ± sd	8.14 ± 0.30	8.13 ± 0.30	8.14 ± 0.30	8.12 ± 0.30	8.14 ± 0.20	8.13 ± 0.30	8.14 ± 0.30
median (min; max)	8.12 (7.31; 9.20)	8.12 (7.31; 9.20)	8.13 (7.48; 8.90)	8.12 (7.31; 8.76)	8.15 (7.54; 8.90)	8.10 (7.33; 9.20)	8.12 (7.48; 8.84)
**TISSUE FACTOR (TF)**							
mean ± sd	6.37 ± 0.30	6.34 ± 0.30	6.39 ± 0.30	6.35 ± 0.30	6.40 ± 0.30	6.34 ± 0.30	6.37 ± 0.30
median (min; max)	6.36 (5.42; 7.59)	6.35 (5.42; 7.28)	6.37 (5.55; 7.59)	6.36 (5.51; 7.17)	6.39 (5.55; 7.59)	6.35 (5.42; 7.28)	6.36 (5.57; 7.19)
**INTERLEUKIN-1 RECEPTOR-LIKE 2 (IL1RL2)**							
mean ± sd	5.57 ± 0.40	5.58 ± 0.40	5.56 ± 0.40	5.58 ± 0.40	5.57 ± 0.40	5.57 ± 0.50	5.55 ± 0.50
median (min; max)	5.58 (4.15; 6.94)	5.60 (4.17; 6.86)	5.57 (4.15; 6.94)	5.60 (4.39; 6.86)	5.56 (4.51; 6.71)	5.60 (4.17; 6.82)	5.61 (4.15; 6.94)
**PLATELED-DERIVED GROWTH FACTOR SUBUNIT B (PDGF subunit B)**							
mean ± sd	8.77 ± 1.00	8.75 ± 1.00	8.79 ± 1.00	8.72 ± 1.00	8.75 ± 1.00	8.77 ± 1.00	8.82 ± 1.00
median (min; max)	8.69 (6.12; 11.70)	8.71 (6.21; 11.70)	8.68 (6.12; 11.62)	8.67 (6.24; 11.59)	8.59 (6.12; 11.62)	8.73 (6.21; 11.70)	8.79 (6.40; 11.05)
**INTERLEUKIN-27 (IL27)**							
mean ± sd	6.45 ± 0.30	6.43 ± 0.30	6.47 ± 0.30	6.41 ± 0.30	6.45 ± 0.30	6.45 ± 0.40	6.50 ± 0.40
median (min; max)	6.46 (5.38; 7.66)	6.45 (5.38; 7.66)	6.46 (5.56; 7.59)	6.44 (5.38; 7.43)	6.45 (5.56; 7.25)	6.47 (5.42; 7.66)	6.48 (5.65; 7.59)
**INTERLEUKIN-17D (IL17D)**							

mean ± sd	3.36 ± 0.50	3.36 ± 0.50	3.36 ± 0.50	3.36 ± 0.40	3.36 ± 0.40	3.36 ± 0.50	3.36 ± 0.60
median (min; max)	3.33 (1.89; 9.88)	3.34 (1.89; 9.13)	3.32 (2.27; 9.88)	3.37 (1.89; 5.58)	3.33 (2.27; 5.05)	3.32 (2.44; 9.13)	3.31 (2.57; 9.88)
**C-X-C MOTIF CHEMOKINE LIGAND 1 (CXCL1)**							
mean ± sd	8.78 ± 0.80	8.76 ± 0.70	8.81 ± 0.80	8.75 ± 0.70	8.79 ± 0.80	8.77 ± 0.80	8.83 ± 0.80
median (min; max)	8.73 (6.89; 11.46)	8.72 (6.93; 11.46)	8.74 (6.89; 11.44)	8.75 (7.17; 11.46)	8.71 (7.04; 11.07)	8.68 (6.93; 11.26)	8.82 (6.89; 11.44)
**LECTIN-LIKE OXIDIZED LDL RECEPTOR (LOX1)**							
mean ± sd	7.15 ± 0.80	7.14 ± 0.80	7.17 ± 0.80	7.11 ± 0.80	7.12 ± 0.90	7.16 ± 0.80	7.21 ± 0.80
median (min; max)	6.95 (5.62; 9.59)	6.95 (5.69; 9.59)	6.96 (5.62; 9.54)	6.95 (5.69; 9.59)	6.89 (5.62; 9.51)	6.95 (5.70; 9.56)	7.01 (5.71; 9.54)
**GALECTIN-9 (Gal9)**							
mean ± sd	8.55 ± 0.30	8.52 ± 0.30	8.57 ± 0.30	8.51 ± 0.30	8.54 ± 0.30	8.53 ± 0.30	8.61 ± 0.30
median (min; max)	8.53 (7.69; 9.91)	8.51 (7.69; 9.91)	8.54 (7.71; 9.57)	8.49 (7.75; 9.91)	8.52 (7.89; 9.47)	8.52 (7.69; 9.31)	8.59 (7.71; 9.57)
**GASTRIC INTRINSIC FACTOR (GIF)**							
mean ± sd	8.91 ± 1.10	8.86 ± 1.10	8.96 ± 1.00	8.81 ± 1.10	8.88 ± 1.10	8.91 ± 1.00	9.03 ± 1.00
median (min; max)	8.88 (3.79; 13.05)	8.82 (4.87; 13.05)	8.95 (3.79; 12.94)	8.72 (4.87; 13.05)	8.89 (4.78; 12.55)	8.88 (5.28; 12.83)	9.09 (3.79; 12.94)
**STEM CELL FACTOR (SCF)**							

mean ± sd	10.28 ± 0.00	10.26 ± 0.00	10.30 ± 0.00	10.26 ± 0.00	10.27 ± 0.00	10.27 ± 0.00	10.32 ± 0.00
median (min; max)	10.33 (8.37; 11.19)	10.30 (8.45; 11.19)	10.36 (8.37; 11.06)	10.32 (8.45; 11.19)	10.34 (8.37; 11.06)	10.28 (8.84; 10.99)	10.41 (8.98; 11.03)
**INTERLEUKIN-18 (IL18)**							
mean ± sd	8.88 ± 0.60	8.86 ± 0.60	8.89 ± 0.60	8.88 ± 0.50	8.87 ± 0.50	8.84 ± 0.60	8.92 ± 0.60
median (min; max)	8.87 (6.87; 10.82)	8.87 (6.87; 10.82)	8.87 (7.47; 10.48)	8.88 (7.52; 10.67)	8.84 (7.67; 10.08)	8.84 (6.87; 10.82)	8.87 (7.47; 10.48)
**FIBROBLAST GROWTH FACTOR 21 (FGF21)**							
mean ± sd	8.00 ± 1.00	7.90 ± 1.40	8.10 ± 1.40	7.90 ± 1.30	8.07 ± 1.40	7.90 ± 1.50	8.12 ± 1.40
median (min; max)	7.79 (4.76; 14.93)	7.70 (4.81; 14.68)	7.99 (4.76; 14.93)	7.75 (5.26; 12.65)	8.01 (5.03; 13.74)	7.61 (4.81; 14.68)	7.97 (4.76; 14.93)
**POLYMERIC IMMUNOGLOBULIN RECEPTOR (PlgR)**							
mean ± sd	3.35 ± 0.10	3.33 ± 0.10	3.36 ± 0.10	3.33 ± 0.10	3.35 ± 0.10	3.34 ± 0.10	3.36 ± 0.10
median (min; max)	3.34 (2.76; 3.73)	3.33 (2.94; 3.65)	3.35 (2.76; 3.73)	3.32 (2.94; 3.63)	3.35 (2.78; 3.70)	3.33 (2.94; 3.65)	3.36 (2.76; 3.73)
**RECEPTOR FOR ADVANCED GLYCOSYLATION END PRODUCTS (RAGE)**							
mean ± sd	13.09 ± 0.00	13.05 ± 0.00	13.13 ± 0.00	13.04 ± 0.00	13.10 ± 0.00	13.06 ± 0.00	13.15 ± 0.00
median (min; max)	13.09 (11.73; 14.34)	13.07 (11.73; 14.12)	13.14 (11.96; 14.34)	13.06 (12.00; 13.96)	13.09 (11.96; 14.19)	13.07 (11.73; 14.12)	13.20 (12.02; 14.34)
**SUPEROXIDE DISMUTASE, MITOCHONDIRAL (SOD2)**							

mean ± sd	9.67 ± 0.10	9.66 ± 0.10	9.67 ± 0.10	9.66 ± 0.10	9.68 ± 0.10	9.66 ± 0.10	9.66 ± 0.10
median (min; max)	9.67 (9.13; 10.02)	9.66 (9.13; 10.02)	9.67 (9.21; 9.96)	9.67 (9.18; 9.96)	9.68 (9.24; 9.96)	9.66 (9.13; 10.02)	9.67 (9.21; 9.96)
**CHYMOTRYPSIN C (CTRC)**							
mean ± sd	10.82 ± 1.00	10.83 ± 1.00	10.81 ± 1.00	10.79 ± 1.00	10.76 ± 1.00	10.87 ± 1.00	10.87 ± 1.00
median (min; max)	10.88 (8.26; 12.99)	10.90 (8.29; 12.72)	10.86 (8.26; 12.99)	10.91 (8.29; 12.72)	10.80 (8.36; 12.49)	10.88 (8.51; 12.17)	10.92 (8.26; 12.99)
**FIBROBLAST GROWTH FACTOR 23 (FGF23)**							
mean ± sd	3.94 ± 0.60	3.91 ± 0.60	3.98 ± 1.00	3.90 ± 0.60	3.93 ± 0.60	3.91 ± 0.60	4.03 ± 1.00
median (min; max)	3.87 (1.99; 6.85)	3.83 (2.48; 6.48)	3.90 (1.99; 6.85)	3.83 (2.67; 6.48)	3.88 (2.73; 6.65)	3.83 (2.48; 6.21)	3.93 (1.99; 6.85)
**SPONDIN-2 (SPON2)**							
mean ± sd	9.81 ± 0.20	9.77 ± 0.20	9.85 ± 0.20	9.77 ± 0.20	9.84 ± 0.20	9.78 ± 0.20	9.86 ± 0.20
median (min; max)	9.82 (9.28; 10.31)	9.78 (9.30; 10.29)	9.85 (9.28; 10.31)	9.77 (9.31; 10.24)	9.84 (9.40; 10.26)	9.79 (9.30; 10.29)	9.86 (9.28; 10.31)
**GROWTH HORMONE (GH)**							
mean ± sd	8.81 ± 2.00	8.64 ± 1.90	8.98 ± 2.00	8.77 ± 1.90	8.82 ± 2.00	8.51 ± 1.90	9.13 ± 2.00
median (min; max)	8.91 (3.76; 12.17)	8.66 (3.76; 12.14)	9.23 (4.23; 12.17)	8.87 (4.07; 11.83)	8.91 (4.23; 11.99)	8.63 (3.76; 12.14)	9.45 (4.24; 12.17)
**FOLLISTATIN (FS)**							

mean ± sd	9.69 ± 0.60	9.74 ± 0.60	9.64 ± 0.50	9.78 ± 0.60	9.63 ± 0.60	9.70 ± 0.60	9.64 ± 0.50
median (min; max)	9.68 (8.03; 11.99)	9.72 (8.13; 11.99)	9.65 (8.03; 11.24)	9.74 (8.28; 11.99)	9.67 (8.03; 11.24)	9.70 (8.13; 11.21)	9.64 (8.45; 11.00)
**LACTOYLGLUTATHIONE LYASE (GLO1)**							
mean ± sd	7.38 ± 0.70	7.38 ± 0.70	7.38 ± 0.70	7.39 ± 0.70	7.36 ± 0.70	7.37 ± 0.70	7.40 ± 0.70
median (min; max)	7.34 (5.51; 9.98)	7.32 (5.63; 9.98)	7.36 (5.51; 9.73)	7.30 (6.05; 9.62)	7.34 (5.51; 9.46)	7.33 (5.63; 9.98)	7.37 (5.55; 9.73)
**SLAM FAMILY MEMBER 5 (CD84)**							
mean ± sd	5.27 ± 0.40	5.26 ± 0.40	5.28 ± 0.40	5.27 ± 0.40	5.28 ± 0.40	5.24 ± 0.40	5.28 ± 0.40
median (min; max)	5.24 (4.15; 7.23)	5.23 (4.15; 7.16)	5.28 (4.29; 7.23)	5.25 (4.41; 7.16)	5.29 (4.29; 6.77)	5.22 (4.15; 7.07)	5.23 (4.30; 7.23)
**PAPPALYSIN-1 (PAPPA)**							
mean ± sd	2.12 ± 0.30	2.12 ± 0.30	2.13 ± 0.30	2.10 ± 0.30	2.11 ± 0.30	2.14 ± 0.40	2.14 ± 0.30
median (min; max)	2.06 (1.77; 3.96)	2.04 (1.77; 3.79)	2.07 (1.77; 3.96)	2.04 (1.77; 2.93)	2.05 (1.78; 3.05)	2.04 (1.77; 3.79)	2.11 (1.77; 3.96)
**SERPIN A12 (SERPINA12)**							
mean ± sd	3.59 ± 1.10	3.56 ± 1.00	3.62 ± 1.10	3.53 ± 1.00	3.60 ± 1.10	3.58 ± 1.10	3.64 ± 1.10
median (min; max)	3.44 (1.35; 8.19)	3.43 (1.48; 7.93)	3.48 (1.35; 8.19)	3.45 (1.58; 7.80)	3.47 (1.35; 8.19)	3.42 (1.48; 7.93)	3.48 (1.65; 7.89)
**RENIN (REN)**							

mean ± sd	8.11 ± 1.00	7.92 ± 1.00	8.31 ± 1.00	7.84 ± 1.00	7.97 ± 1.00	7.99 ± 1.00	8.63 ± 0.90
median (min; max)	8.15 (4.60; 10.36)	7.89 (4.60; 10.27)	8.34 (5.15; 10.36)	7.80 (4.60; 10.27)	7.97 (5.15; 10.36)	7.99 (5.41; 10.20)	8.65 (6.22; 10.30)
**2,4-DIENOYL-COA-REDUCTASE, MITOCHONDRIAL (DECR1)**							
mean ± sd	4.52 ± 1.10	4.68 ± 1.10	4.36 ± 1.00	4.62 ± 1.10	4.39 ± 1.00	4.73 ± 1.10	4.33 ± 1.10
median (min; max)	4.34 (2.45; 10.67)	4.52 (2.45; 10.67)	4.16 (2.51; 9.12)	4.40 (2.67; 10.67)	4.17 (2.51; 9.12)	4.62 (2.45; 9.87)	4.11 (2.53; 8.55)
**TYROSINE-PROTEIN KINASE MER (MERTK)**							
mean ± sd	6.97 ± 0.00	6.95 ± 0.40	7.00 ± 0.00	6.93 ± 0.40	6.97 ± 0.00	6.96 ± 0.00	7.02 ± 0.00
median (min; max)	6.95 (4.78; 8.55)	6.93 (4.78; 8.12)	6.97 (4.84; 8.55)	6.95 (5.99; 8.05)	6.95 (5.60; 7.85)	6.90 (4.78; 8.12)	6.99 (4.84; 8.55)
**KIDNEY INJURY MOLECULE 1 (KIM1)**							
mean ± sd	9.05 ± 0.80	9.01 ± 1.00	9.10 ± 0.80	9.02 ± 1.00	9.04 ± 1.00	9.01 ± 1.00	9.14 ± 0.80
median (min; max)	8.96 (6.79; 11.96)	8.92 (6.79; 11.40)	9.03 (7.01; 11.96)	8.92 (7.46; 11.30)	8.95 (7.01; 11.96)	8.92 (6.79; 11.40)	9.08 (7.35; 11.44)
**THROMBOSPONDIN-2 (THBS2)**							
mean ± sd	5.96 ± 0.00	5.95 ± 0.00	5.97 ± 0.00	5.95 ± 0.20	5.97 ± 0.00	5.96 ± 0.00	5.97 ± 0.00
median (min; max)	5.96 (5.40; 6.93)	5.95 (5.40; 6.93)	5.96 (5.57; 6.63)	5.94 (5.40; 6.43)	5.97 (5.57; 6.63)	5.96 (5.43; 6.93)	5.95 (5.57; 6.54)
**THROMBOMODULIN (TM)**							

mean ± sd	11.17 ± 0.00	11.14 ± 0.00	11.19 ± 0.00	11.14 ± 0.00	11.19 ± 0.00	11.15 ± 0.00	11.20 ± 0.00
median (min; max)	11.15 (9.94; 12.31)	11.14 (10.16; 12.20)	11.20 (9.94; 12.31)	11.15 (10.16; 11.83)	11.21 (9.94; 11.99)	11.12 (10.16; 12.20)	11.20 (10.33; 12.31)
**V-SET AND IMMUNOGLOBULIN DOMAIN-CONTAINING PROTEIN 2 (VSIG2)**							
mean ± sd	4.93 ± 0.70	4.85 ± 0.60	5.01 ± 1.00	4.87 ± 0.60	4.99 ± 1.00	4.84 ± 0.60	5.03 ± 1.00
median (min; max)	4.82 (3.07; 8.24)	4.74 (3.07; 7.32)	4.92 (3.23; 8.24)	4.74 (3.07; 7.32)	4.88 (3.57; 8.24)	4.75 (3.37; 6.77)	4.92 (3.23; 7.43)
**ALPHA-1-MICROGLOBULIN/BIKUNIN PRECURSOR (AMBP)**							
mean ± sd	8.02 ± 0.00	8.00 ± 0.00	8.04 ± 0.00	8.01 ± 0.00	8.02 ± 0.00	7.99 ± 0.00	8.06 ± 0.20
median (min; max)	8.02 (7.25; 8.55)	8.01 (7.28; 8.55)	8.04 (7.25; 8.54)	8.02 (7.52; 8.55)	8.02 (7.44; 8.51)	8.00 (7.28; 8.43)	8.08 (7.25; 8.54)
**PROLARGIN (PRELP)**							
mean ± sd	8.34 ± 0.20	8.33 ± 0.20	8.35 ± 0.20	8.34 ± 0.20	8.35 ± 0.20	8.32 ± 0.20	8.36 ± 0.20
median (min; max)	8.35 (7.24; 9.10)	8.35 (7.61; 8.83)	8.35 (7.24; 9.10)	8.36 (7.68; 8.83)	8.35 (7.24; 8.84)	8.34 (7.61; 8.83)	8.36 (7.34; 9.10)
**HEME OXYGENASE 1 (HO1)**							
mean ± sd	12.48 ± 0.00	12.46 ± 0.00	12.49 ± 0.00	12.48 ± 0.00	12.52 ± 0.00	12.44 ± 0.00	12.47 ± 0.00
median (min; max)	12.51 (10.18; 13.66)	12.51 (10.35; 13.65)	12.52 (10.18; 13.66)	12.52 (10.89; 13.47)	12.54 (10.18; 13.38)	12.47 (10.35; 13.65)	12.48 (10.22; 13.66)
**LYMPHOTACTIN (XCL1)**							

mean ± sd	5.68 ± 0.60	5.67 ± 0.60	5.69 ± 0.60	5.71 ± 0.50	5.71 ± 0.50	5.65 ± 0.60	5.68 ± 0.70
median (min; max)	5.66 (3.95; 9.33)	5.66 (3.95; 9.33)	5.66 (4.04; 9.33)	5.70 (4.24; 7.03)	5.68 (4.17; 6.95)	5.63 (3.95; 9.33)	5.61 (4.04; 9.33)
**PRO-INTERLEUKIN-16 (IL16)**							
mean ± sd	7.55 ± 0.50	7.55 ± 0.50	7.55 ± 0.50	7.56 ± 0.50	7.55 ± 0.50	7.54 ± 0.50	7.54 ± 0.50
median (min; max)	7.55 (5.05; 9.86)	7.55 (5.05; 9.86)	7.53 (5.31; 9.23)	7.57 (5.67; 9.86)	7.57 (6.10; 9.03)	7.55 (5.05; 9.40)	7.53 (5.31; 9.23)
**SORTILIN (SORT1)**							
mean ± sd	9.15 ± 0.30	9.15 ± 0.30	9.14 ± 0.30	9.15 ± 0.30	9.14 ± 0.20	9.15 ± 0.30	9.15 ± 0.30
median (min; max)	9.13 (8.33; 10.14)	9.13 (8.33; 10.14)	9.13 (8.39; 10.05)	9.14 (8.47; 10.14)	9.15 (8.58; 9.81)	9.13 (8.33; 10.03)	9.12 (8.39; 10.05)
**CARCINOEMBRYONIC ANTIGEN-RELATED CELL ADHESION MOLECULE (CEACAM8)**							
mean ± sd	5.06 ± 0.70	5.03 ± 1.00	5.09 ± 0.80	4.99 ± 1.00	4.99 ± 1.00	5.07 ± 0.70	5.18 ± 0.80
median (min; max)	4.93 (3.48; 8.59)	4.90 (3.48; 8.59)	4.94 (3.53; 8.30)	4.90 (3.48; 8.59)	4.91 (3.53; 8.14)	4.92 (3.68; 8.28)	5.06 (3.70; 8.30)
**PENTRAXIN-RELATED PROTEIN PTX3 (PTX3)**							
mean ± sd	4.21 ± 0.50	4.28 ± 0.40	4.14 ± 0.50	4.28 ± 0.40	4.13 ± 0.50	4.27 ± 0.40	4.15 ± 0.50
median (min; max)	4.18 (2.79; 6.78)	4.26 (3.10; 5.81)	4.11 (2.79; 6.78)	4.26 (3.20; 5.81)	4.09 (2.95; 6.77)	4.25 (3.10; 5.53)	4.16 (2.79; 6.78)
**P-SELECTIN GLYCOPROTEIN LIGAND (PSGL1)**							

mean ± sd	4.70 ± 0.30	4.71 ± 0.30	4.69 ± 0.30	4.73 ± 0.30	4.72 ± 0.30	4.68 ± 0.30	4.67 ± 0.30
median (min; max)	4.69 (3.51; 5.61)	4.70 (3.61; 5.61)	4.68 (3.51; 5.49)	4.73 (3.66; 5.43)	4.70 (3.87; 5.38)	4.68 (3.61; 5.61)	4.66 (3.51; 5.49)
**C-C MOTIF CHEMOKINE LIGAND 17 (CCL17)**							
mean ± sd	8.61 ± 0.70	8.61 ± 0.70	8.61 ± 0.70	8.62 ± 0.70	8.61 ± 0.70	8.60 ± 0.70	8.62 ± 0.60
median (min; max)	8.57 (6.84; 11.77)	8.56 (6.84; 11.77)	8.57 (6.97; 11.07)	8.60 (6.84; 11.72)	8.57 (6.97; 11.07)	8.54 (7.16; 11.77)	8.57 (7.17; 10.32)
**C-C MOTIF CHEMOKINE LIGAND 3 (CCL3)**							
mean ± sd	7.31 ± 0.70	7.26 ± 0.60	7.36 ± 0.70	7.25 ± 0.50	7.32 ± 0.60	7.27 ± 0.70	7.40 ± 0.80
median (min; max)	7.26 (5.86; 13.78)	7.21 (5.91; 12.83)	7.30 (5.86; 13.78)	7.23 (5.91; 9.09)	7.31 (5.86; 9.97)	7.18 (6.04; 12.83)	7.30 (6.12; 13.78)
**MATRIX METALLOPROTEINASE 7 (MMP7)**							
mean ± sd	11.56 ± 0.00	11.53 ± 0.00	11.59 ± 0.00	11.52 ± 0.00	11.55 ± 0.00	11.54 ± 0.00	11.62 ± 0.00
median (min; max)	11.57 (10.69; 12.25)	11.54 (10.69; 12.25)	11.60 (10.74; 12.23)	11.53 (10.78; 12.12)	11.56 (10.99; 12.04)	11.56 (10.69; 12.25)	11.64 (10.74; 12.23)
**LOW AFFINITY IMMUNOGLOBULIN GAMMA FC REGION RECEPTOR II-B (IgG Fc receptor IIB)**							
mean ± sd	4.37 ± 0.90	4.35 ± 0.90	4.39 ± 0.90	4.32 ± 0.90	4.36 ± 0.90	4.37 ± 0.90	4.41 ± 1.00
median (min; max)	4.49 (1.38; 6.60)	4.50 (1.50; 6.25)	4.49 (1.38; 6.60)	4.45 (2.11; 6.25)	4.48 (2.24; 6.27)	4.56 (1.50; 6.13)	4.49 (1.38; 6.60)
**MELUSIN (ITGB1BP2)**							

mean ± sd	3.21 ± 0.80	3.22 ± 0.80	3.20 ± 0.80	3.14 ± 0.80	3.18 ± 0.90	3.30 ± 0.90	3.22 ± 0.70
median (min; max)	2.98 (2.36; 7.36)	2.97 (2.36; 7.36)	3.02 (2.36; 6.91)	2.91 (2.36; 7.36)	2.93 (2.36; 6.91)	3.11 (2.37; 6.61)	3.12 (2.36; 5.31)
**DECORIN (DCN)**							
mean ± sd	5.46 ± 0.30	5.43 ± 0.20	5.49 ± 0.30	5.44 ± 0.20	5.49 ± 0.20	5.42 ± 0.30	5.49 ± 0.30
median (min; max)	5.43 (4.87; 6.92)	5.40 (4.87; 6.89)	5.46 (4.94; 6.92)	5.41 (4.93; 6.60)	5.45 (5.02; 6.42)	5.40 (4.87; 6.89)	5.48 (4.94; 6.92)
**DICKKOPF-RELATED PROTEIN 1 (Dkk1)**							
mean ± sd	8.10 ± 0.50	8.09 ± 0.50	8.11 ± 0.50	8.08 ± 0.50	8.08 ± 0.50	8.11 ± 0.50	8.13 ± 0.50
median (min; max)	8.06 (6.57; 10.44)	8.06 (6.81; 10.44)	8.06 (6.57; 10.28)	8.06 (6.81; 10.04)	8.00 (6.80; 9.77)	8.07 (6.91; 10.44)	8.11 (6.57; 10.28)
**LIPOPROTEIN LIPASE (LPL)**							
mean ± sd	10.20 ± 1.00	10.19 ± 1.00	10.22 ± 1.00	10.19 ± 1.00	10.20 ± 1.00	10.18 ± 1.00	10.23 ± 1.00
median (min; max)	10.29 (7.87; 11.17)	10.29 (8.22; 11.08)	10.29 (7.87; 11.17)	10.30 (8.22; 11.03)	10.29 (8.18; 11.16)	10.29 (8.24; 11.08)	10.31 (7.87; 11.17)
**PROSTATIN (PRSS8)**							
mean ± sd	9.57 ± 0.40	9.54 ± 0.40	9.61 ± 0.40	9.55 ± 0.40	9.60 ± 0.40	9.53 ± 0.40	9.61 ± 0.40
median (min; max)	9.60 (8.22; 10.63)	9.57 (8.22; 10.54)	9.62 (8.31; 10.63)	9.59 (8.52; 10.54)	9.63 (8.31; 10.34)	9.56 (8.22; 10.32)	9.62 (8.43; 10.63)
**AGOUTI-RELATED PROTEIN (AGRP)**							

mean ± sd	6.21 ± 0.40	6.19 ± 0.40	6.23 ± 0.40	6.20 ± 0.30	6.22 ± 0.40	6.18 ± 0.40	6.24 ± 0.40
median (min; max)	6.20 (4.82; 7.73)	6.20 (4.83; 7.24)	6.21 (4.82; 7.73)	6.19 (5.31; 7.24)	6.23 (4.82; 7.34)	6.20 (4.83; 7.18)	6.20 (5.00; 7.73)
**PROHEPARIN-BINDING EGF-LIKE GROWTH FACTOR (HB-EGF)**							
mean ± sd	4.55 ± 0.30	4.53 ± 0.30	4.57 ± 0.30	4.54 ± 0.30	4.56 ± 0.30	4.52 ± 0.30	4.58 ± 0.30
median (min; max)	4.59 (2.22; 5.34)	4.56 (2.22; 5.34)	4.61 (2.39; 5.26)	4.57 (2.99; 5.34)	4.60 (2.39; 5.13)	4.55 (2.22; 5.20)	4.62 (2.44; 5.26)
**GROWTH/DIFFERENTIATION FACTOR 2 (GDF2)**							
mean ± sd	8.25 ± 0.40	8.22 ± 0.40	8.28 ± 0.40	8.21 ± 0.40	8.28 ± 0.40	8.23 ± 0.40	8.28 ± 0.40
median (min; max)	8.25 (6.01; 10.01)	8.24 (6.40; 10.01)	8.28 (6.01; 9.77)	8.22 (7.19; 9.43)	8.25 (7.25; 9.77)	8.24 (6.40; 10.01)	8.30 (6.01; 9.49)
**FATTY ACID BINDING PROTEIN 2 (FABP2)**							
mean ± sd	9.05 ± 0.80	8.98 ± 1.00	9.12 ± 0.80	9.05 ± 0.70	9.14 ± 0.80	8.92 ± 0.80	9.10 ± 0.90
median (min; max)	9.04 (6.67; 11.74)	8.98 (6.67; 11.50)	9.11 (6.71; 11.74)	9.00 (6.94; 11.50)	9.13 (7.16; 11.74)	8.97 (6.67; 10.89)	9.10 (6.71; 11.68)
**THROMBOPOIETIN (THPO)**							
mean ± sd	3.10 ± 0.30	3.07 ± 0.30	3.12 ± 0.30	3.10 ± 0.30	3.12 ± 0.30	3.04 ± 0.00	3.12 ± 0.30
median (min; max)	3.10 (2.01; 4.21)	3.09 (2.25; 4.12)	3.12 (2.01; 4.21)	3.12 (2.30; 4.11)	3.12 (2.01; 4.19)	3.03 (2.25; 4.12)	3.11 (2.37; 4.21)
**MACROPHAGE RECEPTOR WITH COLLAGENOUS STRUCTURE (MARCO)**							

mean ± sd	7.55 ± 0.20	7.54 ± 0.20	7.55 ± 0.20	7.53 ± 0.20	7.54 ± 0.20	7.55 ± 0.20	7.56 ± 0.20
median (min; max)	7.54 (6.82; 8.21)	7.53 (6.82; 8.18)	7.55 (6.90; 8.21)	7.53 (6.82; 8.11)	7.55 (6.93; 7.96)	7.54 (6.84; 8.18)	7.55 (6.90; 8.21)
**GASTROTROPIN (GT)**							
mean ± sd	2.31 ± 0.60	2.25 ± 0.60	2.37 ± 0.70	2.30 ± 0.70	2.40 ± 0.70	2.20 ± 0.60	2.34 ± 0.70
median (min; max)	2.25 (0.61; 5.12)	2.20 (0.85; 4.81)	2.33 (0.61; 5.12)	2.21 (0.98; 4.81)	2.38 (0.65; 5.12)	2.17 (0.85; 4.05)	2.27 (0.61; 4.68)
**BRAIN NATRIURETIC PEPTIDE (BNP)**							
mean ± sd	3.12 ± 1.10	3.13 ± 1.10	3.11 ± 1.10	3.05 ± 1.00	3.21 ± 1.10	3.20 ± 1.20	3.00 ± 1.00
median (min; max)	2.91 (1.61; 6.86)	2.89 (1.61; 6.86)	2.99 (1.62; 6.41)	2.77 (1.61; 6.01)	3.20 (1.62; 6.02)	2.98 (1.61; 6.86)	2.72 (1.64; 6.41)
**MATRIX METALLOPROTEINASE 12 (MMP12)**							
mean ± sd	8.09 ± 0.80	8.09 ± 0.80	8.10 ± 0.80	8.10 ± 0.80	8.10 ± 0.80	8.07 ± 0.80	8.10 ± 0.80
median (min; max)	8.08 (5.72; 11.40)	8.10 (5.72; 10.03)	8.06 (5.74; 11.40)	8.14 (5.72; 10.03)	8.06 (5.74; 10.34)	8.07 (6.01; 9.87)	8.06 (6.04; 11.40)
**ANGIOTENSIN-CONVERTING ENZYME 2 (ACE2)**							
mean ± sd	4.36 ± 0.70	4.35 ± 0.70	4.37 ± 0.70	4.36 ± 0.70	4.37 ± 0.70	4.33 ± 0.70	4.36 ± 0.70
median (min; max)	4.25 (2.98; 6.68)	4.26 (2.98; 6.50)	4.24 (3.06; 6.68)	4.32 (2.98; 6.50)	4.29 (3.06; 6.68)	4.20 (3.03; 6.32)	4.22 (3.15; 6.31)
**PROGRAMMED CELL DEATH 1 LIGAND 2 (PDL2)**							

mean ± sd	4.02 ± 0.00	4.01 ± 0.00	4.04 ± 0.00	3.99 ± 0.00	4.02 ± 0.00	4.03 ± 0.00	4.05 ± 0.00
median (min; max)	4.04 (2.53; 5.56)	4.03 (2.53; 5.56)	4.05 (2.67; 5.27)	4.01 (2.99; 5.04)	4.04 (2.99; 5.04)	4.04 (2.53; 5.56)	4.05 (2.67; 5.27)
**CATHEPSIN L1 (CTSL1)**							
mean ± sd	7.53 ± 0.40	7.49 ± 0.40	7.58 ± 0.40	7.49 ± 0.40	7.56 ± 0.40	7.49 ± 0.40	7.59 ± 0.40
median (min; max)	7.50 (6.53; 8.81)	7.45 (6.53; 8.74)	7.53 (6.69; 8.81)	7.47 (6.53; 8.74)	7.52 (6.69; 8.71)	7.45 (6.79; 8.60)	7.54 (6.77; 8.81)
**OSTEOCLAST-ASSOCIATED IMMUNOGLOBULINE-LIKE RECEPTOR (hOSCAR)**							
mean ± sd	11.78 ± 0.00	11.76 ± 0.00	11.80 ± 0.00	11.74 ± 0.00	11.77 ± 0.00	11.78 ± 0.00	11.83 ± 0.00
median (min; max)	11.79 (10.63; 12.37)	11.77 (10.84; 12.37)	11.82 (10.63; 12.34)	11.74 (10.97; 12.27)	11.78 (10.63; 12.30)	11.80 (10.84; 12.37)	11.85 (10.71; 12.34)
**TUMOR NECROSIS FACTOR RECEPTOR SUPERFAMILY MEMBER 13B (TNFRSF13B)**							
mean ± sd	9.26 ± 0.40	9.24 ± 0.40	9.28 ± 0.40	9.22 ± 0.40	9.25 ± 0.40	9.26 ± 0.40	9.31 ± 0.50
median (min; max)	9.21 (8.25; 11.08)	9.21 (8.25; 10.97)	9.21 (8.31; 11.08)	9.19 (8.25; 10.97)	9.20 (8.31; 11.02)	9.23 (8.30; 10.53)	9.22 (8.34; 11.08)
**TRANSGLUTAMINSASE 2 (TGM2)**							
mean ± sd	6.10 ± 1.00	6.14 ± 1.00	6.07 ± 1.00	6.13 ± 1.00	6.04 ± 1.00	6.15 ± 1.00	6.09 ± 1.10
median (min; max)	5.83 (4.18; 10.81)	5.87 (4.18; 10.42)	5.80 (4.38; 10.81)	5.85 (4.76; 10.42)	5.78 (4.86; 9.48)	5.87 (4.18; 10.17)	5.80 (4.38; 10.81)
**LEPTIN (LEP)**							

mean ± sd	8.03 ± 1.00	7.99 ± 1.00	8.07 ± 1.00	7.97 ± 1.00	8.02 ± 1.00	8.00 ± 1.00	8.12 ± 1.10
median (min; max)	8.08 (3.55; 11.24)	8.02 (4.74; 11.24)	8.15 (3.55; 10.45)	7.99 (5.36; 10.23)	8.08 (5.00; 10.45)	8.06 (4.74; 11.24)	8.27 (3.55; 10.14)
**CARBONIC ANHYDRASE 5A (CA5A)**							
mean ± sd	3.16 ± 1.00	3.19 ± 1.00	3.13 ± 0.90	3.20 ± 0.90	3.18 ± 0.90	3.17 ± 1.00	3.09 ± 0.90
median (min; max)	2.98 (1.81; 8.89)	3.01 (1.81; 8.89)	2.92 (1.84; 6.17)	3.08 (1.82; 6.42)	2.99 (1.84; 5.95)	2.97 (1.81; 8.89)	2.85 (1.84; 6.17)
**HEAT SHOCK 27 KDA PROTEIN (HSP27)**							
mean ± sd	9.57 ± 0.70	9.53 ± 0.70	9.62 ± 0.70	9.53 ± 0.70	9.56 ± 0.70	9.52 ± 0.70	9.67 ± 0.70
median (min; max)	9.60 (7.23; 11.37)	9.51 (7.72; 11.12)	9.67 (7.23; 11.37)	9.48 (7.72; 11.12)	9.61 (7.23; 11.12)	9.58 (7.75; 11.07)	9.71 (7.58; 11.37)
**T-CELL SURFACE GLYCOPROTEIN CD4 (CD4)**							
mean ± sd	5.68 ± 0.30	5.68 ± 0.30	5.69 ± 0.30	5.68 ± 0.30	5.67 ± 0.30	5.68 ± 0.30	5.70 ± 0.30
median (min; max)	5.67 (4.71; 6.74)	5.68 (4.74; 6.67)	5.67 (4.71; 6.74)	5.68 (4.74; 6.67)	5.66 (4.71; 6.74)	5.68 (4.88; 6.58)	5.69 (4.81; 6.72)
**NF-KAPPA-B ESSENTIAL MODULATOR (NEMO)**							
mean ± sd	4.45 ± 0.80	4.48 ± 0.70	4.42 ± 0.80	4.47 ± 0.70	4.41 ± 0.80	4.49 ± 0.80	4.44 ± 0.80
median (min; max)	4.34 (2.60; 8.54)	4.34 (2.66; 8.54)	4.35 (2.60; 7.63)	4.35 (2.90; 8.54)	4.33 (2.60; 7.63)	4.34 (2.66; 7.05)	4.36 (2.79; 7.50)
**VASCULAR ENDOTHELIAL GROWTH FACTOR D (VEGFD)**							

mean ± sd	8.31 ± 0.40	8.31 ± 0.40	8.31 ± 0.40	8.33 ± 0.30	8.36 ± 0.40	8.30 ± 0.40	8.26 ± 0.40
median (min; max)	8.33 (7.04; 9.75)	8.32 (7.17; 9.75)	8.33 (7.04; 9.74)	8.35 (7.41; 9.08)	8.36 (7.12; 9.14)	8.31 (7.17; 9.75)	8.27 (7.04; 9.74)
**POLY [ADP-RIBOSE] POLYMERASE 1 (PARP1)**							
mean ± sd	2.55 ± 0.90	2.60 ± 1.10	2.50 ± 0.80	2.66 ± 1.30	2.54 ± 0.90	2.54 ± 0.70	2.48 ± 0.70
median (min; max)	2.23 (1.80; 7.78)	2.18 (1.80; 7.78)	2.24 (1.80; 6.99)	2.20 (1.80; 7.78)	2.25 (1.80; 6.99)	2.18 (1.81; 4.87)	2.22 (1.80; 5.86)
**HYDROXYACID OXIDASE 1 (HAOX1)**							
mean ± sd	5.82 ± 1.50	5.95 ± 1.00	5.70 ± 1.40	6.03 ± 1.00	5.80 ± 1.40	5.88 ± 1.50	5.59 ± 1.40
median (min; max)	5.66 (2.87; 11.05)	5.74 (3.17; 11.05)	5.53 (2.87; 10.34)	5.85 (3.46; 10.16)	5.71 (2.87; 10.34)	5.66 (3.17; 11.05)	5.37 (3.00; 9.66)

Suppl. Table S3a: Significant associations between the left ventricular mass index (LVMI) and plasma biomarker levels in HFpEF patients at baseline

Biomarker	LVMI [g/m²]	
	b-coefficient (95%-CI)	p-value
BNP	5.79 (2.40 to 9.18)	0.001
MMP12	-4.49 (-8.26 to -0.73)	0.020
DCN	13.38 (1.08 to 25.67)	0.033

Suppl. Table S3b: Significant associations between plasma NT-proBNP concentrations and plasma biomarker levels in HFpEF patients at baseline

Biomarker	NT-proBNP[ng/L]	
	b-coefficient (95%-CI)	p-value
BNP	0.80 (0.70 to 0.90)	<0.001*
VEGFD	1.13 (0.73 to 1.53)	<0.001*
ADM	0.73 (0.45 to 1.01)	<0.001*
PTX3	0.80 (0.47 to 1.13)	<0.001*
HB-EGF	1.03 (0.46 to 1.60)	<0.001*
PRELP	1.22 (0.48 to 1.98)	0.002*
IDUA	-0.37 (-0.63 to -0.12)	0.004*
FS	0.35 (0.11 to 0.59)	0.004*
RAGE	0.52 (0.15 to 0.88)	0.005*
CD4	0.69 (0.20 to 1.81)	0.006*
AGRP	0.59 (0.17 to 1.01)	0.006
DECR1	0.17 (0.04 to 0.30)	0.011
CXCL1	-0.25 (-0.45 to -0.05)	0.015
BOC	0.75 (0.14 to 1.35)	0.016
IL17D	0.39 (0.07 to 0.7)	0.018
FGF23	0.28 (0.04 to 0.52)	0.021
ADAMTS13	-1.30 (-2.42 to -0.19)	0.022
TF	0.60 (0.08 to 1.11)	0.023
GH	0.09 (0.01 to 0.17)	0.026
SPON2	-0.99 (-1.89 to -0.08)	0.033
LPL	0.32 (0.01 to 0.64)	0.044
PlgR	1.22 (0.01 to 2.43)	0.049

Suppl. Table 3c: Significant associations between SF-36 physical functioning scale score and plasma biomarker levels in HFpEF patients at baseline

Biomarker	SF-36 score	
	b-coefficient (95%-CI)	p-value
**Gal9**	-21.87 (-28.96 to -14.78)	<0.001*
**IL1ra**	-9.50 (-12.62 to -6.39)	<0.001*
**TNFRSF11A**	-13.52 (-18.49 to -8.55)	<0.001*
**TRAILR2**	-15.15 (-20.81 to -9.50)	<0.001*
**VSIG2**	-8.56 (-12.16 to -4.95)	<0.001*
**LEP**	-6.46 (-9.26 to -3.66)	<0.001*
**ADM**	-8.76 (-12.72 to -4.81)	<0.001*
**FGF23**	-8.09 (-11.82 to -4.35)	<0.001*
**PGF**	-14.59 (-21.43 to -7.76)	<0.001*
**AMBP**	-25.33 (-37.19 to -13.46)	<0.001*
**SPON2**	-29.59 (-43.73 to -15.45)	<0.001*
**TM**	-14.13 (-20.93 to -7.34)	<0.001*
**IL27**	-14.42 (-21.57 to -7.27)	<0.001*
**TIE2**	-16.71 (-25.01 to -8.40)	<0.001*
**CD84**	-11.34 (-17.07 to -5.60)	<0.001*
**IL6**	-5.33 (-8.05 to -2.61)	<0.001*
**SORT1**	-16.06 (-24.58 to -7.55)	<0.001*
**THBS2**	-23.85 (-36.85 to -10.85)	<0.001*
**CD4**	-13.95 (-21.66 to -6.24)	<0.001*
**PDL2**	-9.34 (-15.13 to -3.54)	0.002*
**REN**	-3.59 (-5.84 to -1.34)	0.002*
**DCN**	-17.31 (-28.18 to -6.45)	0.002*
**FGF21**	-2.58 (-4.21 to -0.95)	0.002*
**KIM1**	-4.39 (-7.23 to -1.55)	0.003*
**GIF**	-3.29 (-5.42 to -1.16)	0.003*
**FS**	-5.98 (-9.91 to -2.06)	0.003*
**MERTK**	-8.80 (-14.81 to -2.80)	0.004*
**Dkk1**	-6.94 (-11.68 to -2.19)	0.004*
**GDF2**	-8.96 (-15.24 to -2.69)	0.005*
**IgG Fc receptor IIb**	-3.54 (-6.03 to -1.04)	0.006*
**TF**	-11.27 (-19.29 to -3.25)	0.006*
**CXCL1**	-4.43 (-7.58 to -1.27)	0.006*
**IL4RA**	-8.44 (-14.55 to -2.34)	0.007*
**MARCO**	-14.61 (-25.21 to -4.01)	0.007*
**PAR1**	-7.95 (-13.72 to -2.18)	0.007*
**HAOX1**	-2.14 (-3.69 to -0.59)	0.007*
**TNFRSF10A**	-6.57 (-11.49 to -1.64)	0.009*
**PRSS27**	-6.79 (-11.88 to -1.70)	0.009*
**CTSL1**	-8.45 (-14.79 to -2.11)	0.009*
**THPO**	-10.02 (-17.58 to -2.47)	0.009*
**PAPPA**	-12.71 (-22.46 to -2.96)	0.011*
**hOSCAR**	-13.84 (-24.70 to -2.98)	0.013*
**MMP7**	-10.58 (-19.05 to -2.11)	0.014*
**CCL3**	-4.37 (-7.95 to -0.78)	0.017*
**XCL1**	-4.94 (-9.00 to -0.89)	0.017*
**STK4**	-3.90 (-7.15 to -0.66)	0.019*

**AGRP**	-7.64 (-14.08 to -1.21)	0.020*
**CA5A**	-2.97 (-5.51 to -0.44)	0.022*
**PTX3**	-6.09 (-11.46 to -0.73)	0.026*
**PRSS8**	-7.65 (-14.40 to -0.89)	0.027*
**ITGB1BP2**	-4.10 (-7.91 to -0.28)	0.036

Linear regression analyses between plasma biomarkers and LVMI (a), plasma NT-proBNP concentrations (b) and the SF-36 physical functioning scale score (c) including age and sex as co-variables were conducted. Proteins with significant associations are ordered by significance in the respective sections; * indicates that statistical significance remains after adjustment for multiple testing.

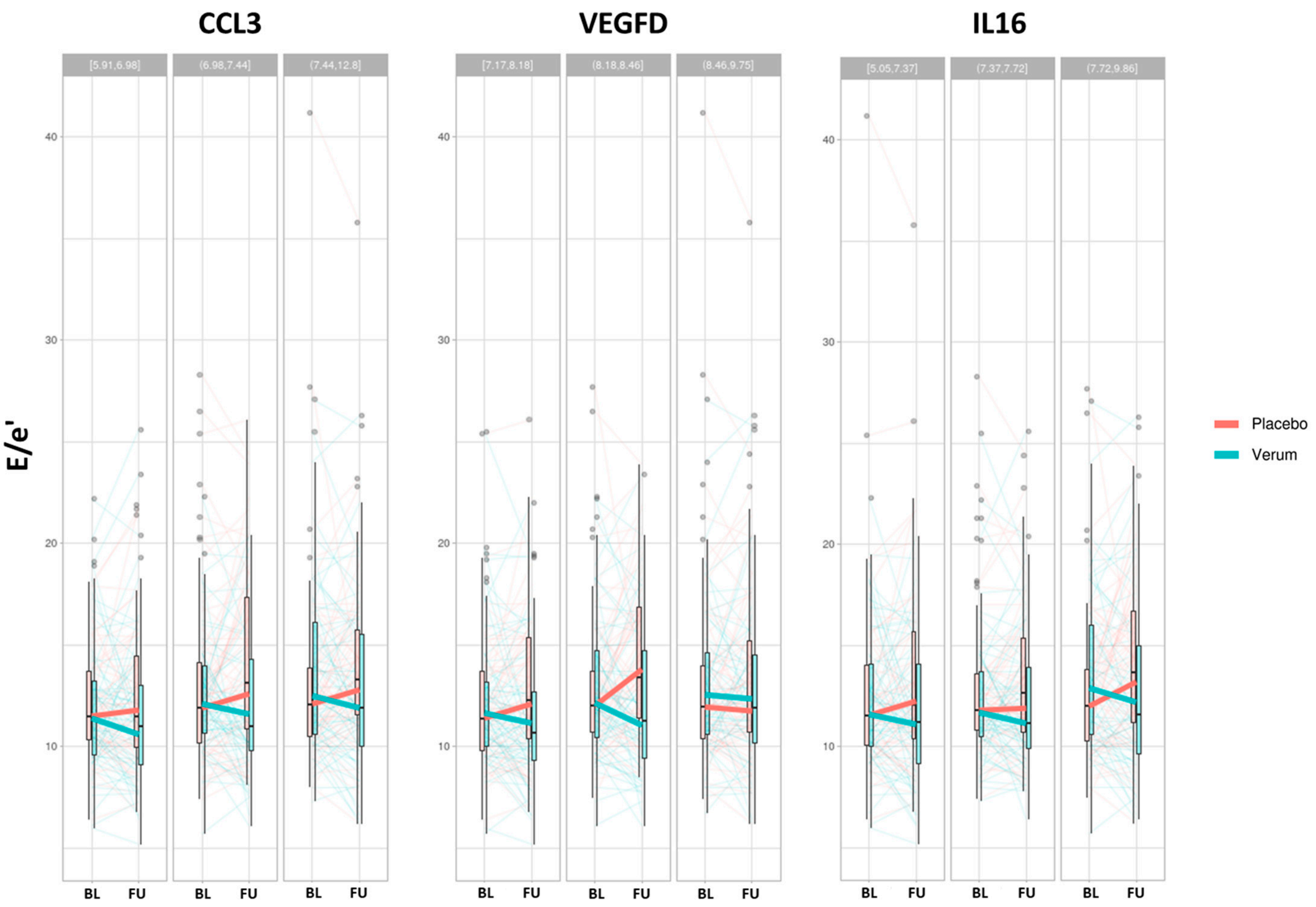

Suppl. Figure S1: Plasma biomarkers with predictive value in regard to spironolactone-mediated effects on relative E/e' changes in HFpEF patients. The effect of the spironolactone treatment (verum vs placebo) on relative changes of E/e' from baseline (BL) to twelve months follow up (FU) was assessed for interaction with BL plasma biomarker expression levels. For visualization purposes, patients were classified by BL expression level tertiles of the respective protein. Only proteins with a significant interaction are shown.

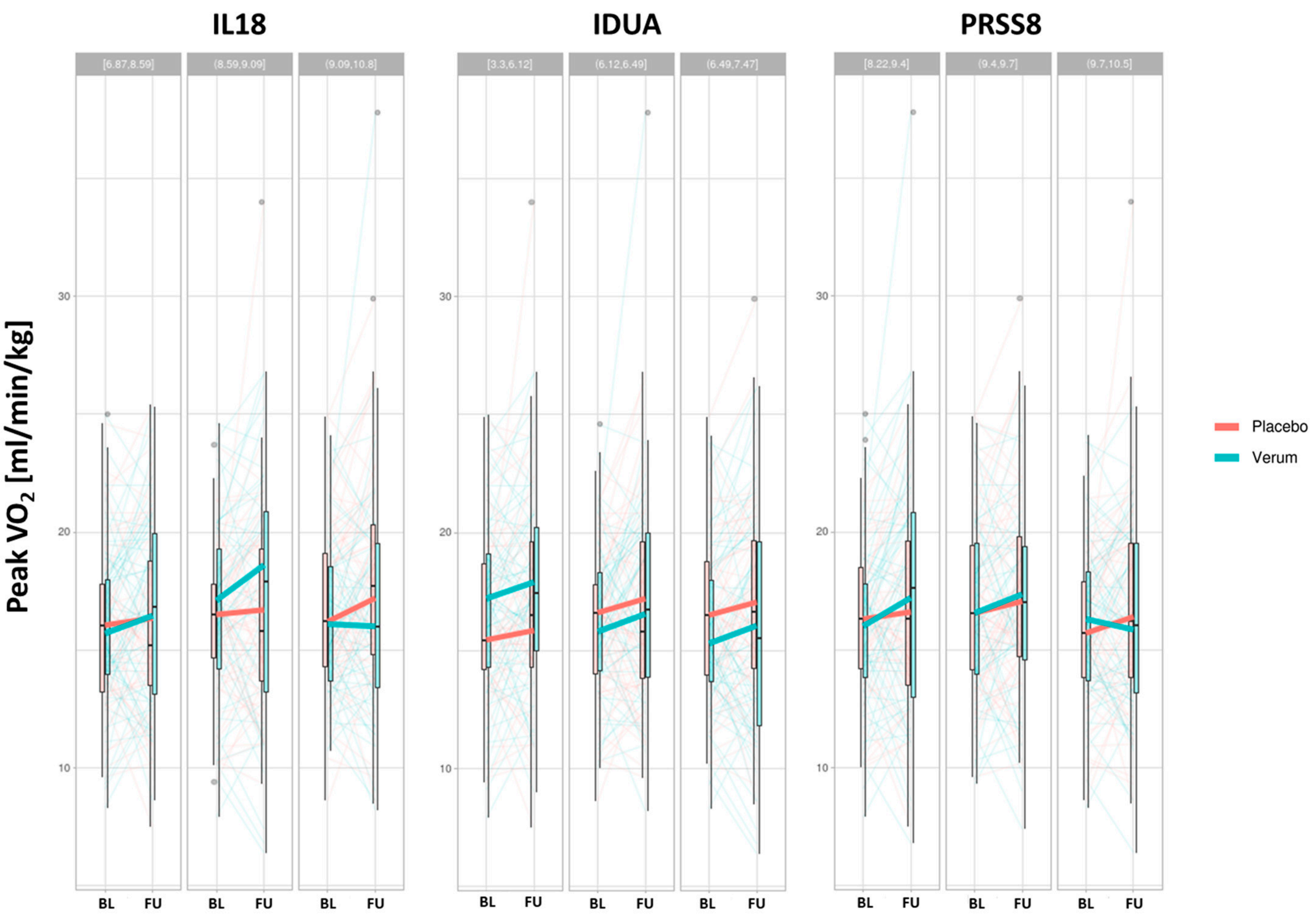

Suppl. Figure S2: Plasma biomarkers with predictive value in regard to spironolactone-mediated effects on relative peak VO_2 changes in HFpEF patients. The effect of the spironolactone treatment (verum vs placebo) on relative changes of peak VO_2 from baseline (BL) to twelve months follow up (FU) was assessed for interaction with BL plasma biomarker expression levels. For visualization purposes, patients were classified by BL expression level tertiles of the respective protein. Only proteins with a significant interaction are shown.
